# Simultaneous detection and partial molecular characterization of five RNA viruses associated with enteric disease in chickens: chicken astrovirus, avian nephritis virus, infectious bronchitis virus, avian rotavirus a and avian orthoreovirus, via multiplex RT–qPCR

**DOI:** 10.3389/fvets.2025.1536420

**Published:** 2025-04-24

**Authors:** Anthony Loor-Giler, Claire Muslin, Silvana Santander-Parra, Dayana Coello, Marcela Robayo-Chico, Antonio Piantino Ferreira, Luis Nunez

**Affiliations:** ^1^Laboratorios de Investigación, Dirección General de InvestigaciónUniversidad de las Américas, Quito, Ecuador; ^2^Facultad de Ingeniería y Ciencias Aplicadas, Carrera de Ingeniería en Biotecnología, Universidad de Las Américas, Quito, Ecuador; ^3^Facultad de Ciencias de la Salud, Carrera de Medicina Veterinaria, Universidad de Las Américas, Quito, Ecuador; ^4^One Health Research Group, Facultad de Ciencias de la Salud, Universidad de Las Americas, Quito, Ecuador; ^5^Laboratory of Avian Diseases, School of Veterinary Medicine and Animal Science, Department of Pathology, University of São Paulo, São Paulo, Brazil

**Keywords:** RT–qPCR, enteric viruses, chickens, RSS, malabsorption syndrome

## Abstract

In the poultry industry, intestinal diseases can lead to significant economic losses due to diarrhea, weight loss and mortality, often linked to viral infections. Chicken astrovirus (CAstV), avian nephritis virus (ANV), infection bronchitis virus (IBV), avian rotavirus A (AvRVA) and avian orthoreovirus (ARV) are key pathogens on this disease including feed malabsorption and runting-stunting syndrome (RSS). This study proposes a multiplex RT–qPCR assay for the simultaneous detection of these five viruses in chickens with enteritis in Ecuador. Primers and hydrolysis probes were designed for the five viruses, along with a synthetic gBlock as a positive control. The method was evaluated for sensitivity, repeatability, and specificity, and 200 jejunal samples were tested. Genome regions of each virus were sequenced, and a phylogenetic analysis confirmed their presence in the samples. The optimized RT-qPCR assay showed efficiency between 98.8–105.9%, with a detection limit of 1 copy/μL. It specifically amplified the five target viruses without cross-reactivity. Among 200 chickens tested, 97% were positive for at least one virus, with ANV (89%) and CAstV (53%) being the most prevalent. Coinfections were common, especially between CAstV and ANV, with three samples positive for all viruses. Sequencing and phylogenetic analysis confirmed the circulation of multiple strains in chickens with enteric disease in Ecuador. This study describes a multiplex RT-qPCR assay for detecting key enteric viruses in Ecuadorian poultry highlighting the high prevalence of astroviruses, emphasizing the impact of coinfections, its possible role in the disease and the importance of improving disease control strategies.

## Introduction

Avian enteric viruses pose a major threat to the poultry industry worldwide because of their high prevalence and potential for rapid transmission, resulting in substantial economic losses. Accurate and timely diagnosis of intestinal infections is crucial for effective disease management, prevention and control (1). Therefore, in recent years, the poultry industry has seen an increased demand for efficient diagnostic methods that can rapidly identify and quantify avian enteric viruses (2). Several types of enteric viruses capable of infecting chickens have been identified, including chicken astrovirus (CAstV), avian nephritis virus (ANV), infectious bronchitis virus (IBV), avian rotavirus A (AvRVA) and avian Orthoreovirus (ARV) (3–5). These viruses are known to cause common intestinal symptoms such as diarrhea, weight loss, weakness, ruffled feathers and decreased egg production. They have been associated with runting-stunting syndrome (RSS), especially in broilers, where halting growth implies massive losses for poultry (6–12). Studies over the years have underscored the severe economic impact of these diseases, with financial losses estimated at approximately $105,000 USD for hatching egg producers and $68,000 USD for hatcheries per 10,000 hens, reflecting the ongoing financial strain on the poultry industry in managing and controlling disease outbreaks (11, 13). While viruses such as laryngotracheitis virus primarily affect the respiratory system (14–16), enteric viruses impact the digestive system, causing severe conditions such as white chick syndrome and malabsorption syndrome, which affect growth and nutrient absorption (12, 17). These viruses can also cause kidney damage and enteritis, and, in severe cases, viral tenositis syndrome, which affects joints and tendons (2, 18). Furthermore, their ability to induce sub-clinical infections represents an additional challenge for detection and management, in contrast to other viruses that present more obvious symptoms (19).

CAstV and ANV are two members of the genus *Avastrovirus* within the *Astroviridae* family that are associated with growth problems, enteritis and kidney lesions in young chickens. CAstV has a positive RNA genome that is approximately 7.5 kb long and contains three open reading frames (ORFs) flanked by two untranslated regions (UTRs) (20, 21). ORF1a and ORF1b encode nonstructural polyproteins, whereas ORF2 encodes capsid proteins. ORF1b has been described as the most conserved region of the genome and has been used several times as a flank for diagnostic methods in addition to 3’UTR, while ORF2 is the more variable region that confers pathogenicity and is used to discriminate genotypes (22). In the case of ANV, ORF2 variation has given it 4 genotypes, while CAstV, which shows high genetic variability, has been classified into 7 subgroups based on pathogenicity (23). This enteric virus is known for its ability to cause subclinical infections, making it a challenging adversary for poultry (24). ANV has a slightly smaller genome than CAstV at approximately 6.8 kb. It is known to cause a wide range of clinical symptoms, with kidney damage as its main characteristic (25, 26). ANV has recently been described in Ecuador (27), where it was identified in 82% of samples analyzed from chickens with enteritis, higher than the 12.6% reported in Brazil in 2018 and the 23% reported in China in 2022 (3, 28).

IBV, a highly contagious virus within the *Gammacoronavirus* genus of the *Coronaviridae* family, affects chickens primarily, causing respiratory, reproductive, renal and digestive diseases. Its genome, approximately 27 kb in length, is flanked by 5′ and 3’ UTRs and includes ORFs encoding nonstructural proteins 1a and 1ab, structural proteins spike (S), envelope (E), membrane (M), and nucleocapsid (N), as well as accessory proteins ORF3 and ORF5 (29, 30). Previous observations have revealed major heterogeneity of the virus in the S1 region of the S gene, leading to its classification into six groups (GI-GVI). According to the analysis on 2016, GI has been identified as the most distributed worldwide, with approximately 27 main lineages (31). Recently, the emergence of new GI lineages concerning their impact on virulence has been hypothesized (32–34). Due to the high genetic variability of this virus, the use of UTRs as a target for molecular methods has been the most viable form of diagnosis (35, 36). Focusing on the respiratory area, IBV has been previously identified in Ecuador in up to 34% of positive samples in 2022 (37). In Brazil, the presence of this virus was detected in association with enteric disease with 58.9% and in China with unreported prevalence (28, 38).

ARV is a non-enveloped, icosahedral virus belonging to the *Reoviridae* family and the genus *Orthoreovirus*. Its genome consists of 10 double-stranded RNA fragments classified according to electrophoretic mobility: L1-L3, of up to 4 kb encoding viral replication proteins, M1-3, of up to 2.4 kb encoding morphogenesis and assembly proteins, and S1-S4, of up to 1.6 kb encoding viral adhesion and host interaction protein (39). The σC gene in the S1 segment is highly variable and used to classify ARV into six genotypes (40). The M1-3 regions have been the most widely used for diagnosis due to their low genetic variability compared to the other regions, in some cases combining more than one for specific genotypes (41). ARV affects poultry, particularly chickens, causing viral arthritis, tenosynovitis, and malabsorption syndrome, impacting joints, tendons, and the intestinal tract (41, 42). AvRVA is another virus in the *Reoviridae* family that specifically belongs to the genus *Rotavirus* (43), has a genome of 11 segments of double-stranded RNA encapsulated in three layers of proteins enconding the structural proteins VP1-VP4, VP6 and VP7, that form the viral capside and host adhesion, and the nonstructural proteins NSP1-NSP6, which are involved in the replication and evasion of immunity (44). Based on variations in the VP6 protein-encoding gene, highly conserved protein of the virion’s internal capsid, rotaviruses (RVs) are classified into eight groups (A to H). AvRVs fall into Groups A, D, F and G, with Group A being the most widely distributed worldwide (45–48). On the other hand, the NSP4 gene, which codes for the viral enterotoxin, is used for specific molecular diagnostic methods covering certain strains, such as those infecting poultry (49). Like ARVs, AvRVs are implicated in growth delay and growth retardation syndrome in poultry. They are associated with both RSS and poult enteritis syndrome (PES) in turkeys (49, 50). Although AvRVA and ARV are recognized as circulating in Ecuador, there is no clear data on the prevalence of these viruses in this region. In Brazil, China and the United States, highly variable prevalences between 0.5 and 20% of chickens with and without enteric disease have been reported (28, 41, 51, 52).

Real-time quantitative polymerase chain reaction (qPCR) has emerged as a powerful tool for sensitive and specific virus detection, offering many advantages, such as quick results, quantified evaluation and the ability to differentiate between viral strains (5, 49, 53, 54). In Ecuador, several outbreaks of enteric disease have been described by poultry farmers, who frequently report outbreaks of intestinal disorders, which are characterized by weak animals with ruffled feathers, cloacal clogging, dwarfism, growth disparity and diarrhea (unpublished data). These enteric diseases have emerged as major challenges for these producers, as no causative bacteria have been isolated or identified, and antimicrobial therapies have proven ineffective. For the detection of ANV and CAstV there is only one duplex qPCR assay (55), and a single assay for ANV (27), plus an endpoint assay including AvRVA (49), which have low detection limits, while for the detection of IBV, ARV single RT-qPCR methods or grouping with other non-enteric viruses have emerged (35, 56). Only for IBV and ARV there are serological methods used for commercial diagnostic for the detection of IBV and ARV, however these are only able to identify genotypes or specific strains of the virus, without covering all versions of this virus (57–61). At the moment, there are no molecular methods that simultaneously detect the presence of these 5 viruses. Therefore, given that they are among the most frequent enteric viruses and the above-mentioned pathological effects, a robust and rapid diagnostic method to identify the possible agents causing this disease is crucial.

This study aimed to develop and explore the application of multiplex RT-qPCR as a cutting edge diagnostic method for avian enteric viruses in chickens. In order to assess its efficacy in chicken samples exhibiting enteritis, necessitating the analysis of these viruses in relation to pertinent statistical variables such as age, location, and coinfections. Finally, to conduct a phylogenetic evaluation of these viruses using conserved genomic segments to validate the accuracy of the detection results obtained through the proposed method. These advances not only enhance our ability to detect these viruses but also provide valuable data for epidemiological studies, helping to formulate effective strategies for controlling and preventing poultry mortality (2, 28, 62). Consequently, a multiplex RT–qPCR assay was developed and used to identify and quantify the presence of the five main RNA viruses associated with intestinal disease, CAstV, ANV, IBV, AvRVA and ARV, enabling accurate diagnosis of the causes of intestinal-associated problems in poultry.

## Methodology

### Sample collection

In this study, 200 jejunum samples from chickens of different age groups, breeds and provinces of Ecuador [Pichincha (112 samples), Imbabura (32 samples), Chimborazo (24 samples) and Tungurahua (32 samples)] with enteric disease were sent to research laboratory located at the Universidad de Las Americas (UDLA) for molecular diagnosis of enteric viruses. According to the medical records of poultry farmers, the animals from which the samples were collected presented signs of intestinal disease, mainly diarrhea, ruffled feathers, dwarfism and weight loss. The chicken intestinal samples were originally collected from deceased animals and transported at 4°C to the laboratory. Upon arrival, aliquots of the jejunum were taken and stored at-20°C until use. All procedures performed in this study were approved by the Committee for the Care and Use of Domestic and Laboratory Animal Resources of the Agency for Regulation and Phytosanitary and Zoosanitary Control of Ecuador (AGROCALIDAD), under number #INT/DA/019.

### Nucleic acid extraction

For the extraction process, approximately 100 mg of jejunum was macerated via stainless steel beads in TissueLyser LT (Qiagen®, United States). The macerated samples were then subjected to RNA extraction via TRIzol reagent (Invitrogen by Life Technologies, Carlsbad, CA, USA) according to the manufacturer’s instructions. The samples were eluted with DEPC-treated water and reverse transcribed. The extracted RNA was stored at −80°C until use. For positives control of DNA virus (ChPV and FAdV-1), a phenol/chloroform based method was used with GT reagent according to previously described protocol (63).

### Primer and hydrolysis probe design

Five primer and hydrolysis probe sets were designed for the simultaneous detection of CAstV, ANV, IBV, AvRVA and ARV (Table 1). All available sequences of the complete CAstV and IBV genomes, the complete ARV M1 segment, the AvRVA segment 10, and all complete and partial ANV genomic sequences were obtained from GenBank and aligned via Clustal Omega with Geneious Prime 2023.0.2 (64). Primers and probes for CAstV, ANV and AvRVA were manually designed using conserved regions of the viral genomes. For IBV and ARV detection, existing primer and probe sets have been adapted to accommodate sequence variations and optimized for multiplexing (35, 56). Primers and probes were designed using an align of a condensate of the sequences uploaded to GenBank for each of the viruses up to 2023, based on regions where little genetic variability has been seen, it is expected that these will remain viable despite the emergence of new strains. Each primer and hydrolysis probe sequence were analyzed for potential dimer formation via the IDT OligoAnalyzer™ tool (65). Additionally, *in silico* BLAST analysis was conducted for each designed sequence to verify specificity, confirming the absence of cross-reactivity.

**Table 1:** Primers and probes used in this study.

Primers and probes	Target	Test	Sequence (5′ – 3′)	Amplicon	Reference
CastV-Fwd	ORF1b + ORF2	RT-qPCR multiplex	GGAGTATGCYGCTGCTGA	106 bp	This study
CastV-Rev			CTTATCGGCCATGCTGCT		
CastV-Probe			FAM- TCGGTCCATCCCTCTACCAGATTTTCTGA/BHQ1		
ANV-Fwd	3’ UTR		GTAAACCACTGGCTGGCT	73 bp	This study
ANV-Rev			TCCTGTACCCTCGATGCTA		
ANV-Probe			HEX- CTACAGCAACTGACTTTCCCGAGGC/BHQ1		
IBV-Fwd	5’ UTR		GCTTTTGAGCCTAGCGTT	150 bp	(35), modified
IBV-Rev			GCTTGAAGCCATGTTGTCA		
IBV-Probe			TEXAS RED- CACCACCAGAACCTGTCACCTC-BHQ2		
ARV-Fwd	Segment M1		GGCCTATCTAGCCACACC	107 bp	(56), modified
ARV-Rev			ACACATCTTGGAGGTCGA		
ARV-Probe			Quasar 670-TGTGCTAGGAGTCGGTTCTCGCATTAC-BHQ2		
AvRVA-Fwd	NSP4		TCTACGTTGTCGAAAGAGGC	95 bp	This study
AvRVA-Rev			CTTGCCACACCCGATCAT		
AvRVA-Probe			CY5-CACCACCAGAACCTGTCACCTC-BHQ2		
Cas Pol 1 F	Orf1b	End point RT-PCR for sequencing	GAY CAR CGA ATG CGR AGR TTG	362 bp	(49)
Cas Pol 1 R			TCA GTG GAA GTG GGK ART CTA		
Anv Pol 1 F	Orf1b		GYT GGG CGC YTC YTT TGA YAC	473 bp	(49)
Anv Pol 1R			CRT TTG CCC KRT ART CTT TRT		
XCE2-	S1		CTC TAT AAA CAC CCT TACA	465 bp	(68)
XCE1+			CAC TGG TAA TTT TTC AGA TGG		
NSP4 F30	NSP4		GGG CGT GCG GAA AGA TGG AGA AC	630 bp	(49)
NSP4 R660			GGG GTT GGG GTA CCA GGG ATT AA		
REO-F	S1		TCM RTC RCA GCG AAG AGA RGT CG	1,114 bp	(69)
REO-R			TCR RTG CCS GTACGC AMG G		

### Standard curve construction

A synthetic double-stranded DNA fragment (gBlocks Gene Fragments, IDT) containing the target genomic sequences of all five viruses (Supplementary material) was used to calibrate the standard curve (66). The gBlock designed was dissolved in 50 μL of UltraPure™ DNase/RNase-Free Distilled Water (Invitrogen) following the manufacturer’s instructions. The solution was subsequently quantified with NanoDrop™ 2000 equipment (Thermo Fisher Scientific, Carlsbad, CA, United States). The obtained DNA concentration was then input into the DNA Copy Number and Dilution Calculator tool (Thermo Fisher Scientific) to determine the required amount of gBlock to generate a standard with 10^8^ copies of genetic material per microliter. Subsequently, serial tenfold dilutions of the DNA standards (gBlock fragment) were performed until 1 copy of genetic material per microliter was reached for the construction of standard curves for the qPCR assays. A tissue (Jejunum) suspension was used as matrix.

### Reverse transcriptase reaction

Prior to the RT-qPCR assay, the RNA samples were subjected to reverse transcriptase (RT) to produce complementary DNA (cDNA). Reverse transcription was performed with 5 μL of extracted RNA via Super Script III (Invitrogen, Waltham, MA, United States) according to the manufacturer’s instructions, with 1 μL of oligo (dT)20 and 1 μL of random hexamer primer. The incubation process was performed with an initial incubation at 25°C for 10 min, followed by a second incubation at 37°C for 50 min and inactivation of the reaction at 70°C for 15 min. The cDNA obtained was either subjected to qPCR or stored in a freezer at −80°C.

### RT–qPCR assay

In this study, the single and multiplex reactions were optimized on the basis of the concentration of primers and hydrolysis probes, as well as the optimal melting temperature for all the targets. The RT–qPCR was carried out in a final volume of 10 μL. To achieve this, TaqPath™ ProAmp™ Multiplex Master Mix (Applied Biosystems Taqman) was used, with a final concentration of 0.2 μM for each primer and 0.1 μM for each probe (Table 1). Additionally, 1 μL of cDNA was added, and UltraPure™ DNase/RNase-Free Distilled Water (Invitrogen) was used to adjust the volume to 10 μL. The RT–qPCR was performed on a CFX96 Touch Real-Time PCR Detection System thermal cycler (Bio-Rad Laboratories, Inc., Hercules, CA 94547, United States) under the following temperature conditions: one cycle at 60°C for 30 s for pre-read, one cycle at 95°C for 5 min for polymerase activation and initial denaturation, foll1owed by 40 cycles at 95°C for 15 s, 61°C for 30 s (Read) and 72°C for 30 s. Following protocol optimization, the analysis of all 200 samples was conducted, incorporating ddH2O as the negative control, a synthetic double-stranded DNA fragment as the positive control, and a non-template control. Each sample was processed in duplicate, including standards for calibration curve. This approach enabled the absolute quantification of all samples.

### Analytical sensitivity and limit of detection and quantification for the RT–qPCR assay

The limit of detection (LoD) and the limit of quantification (LoQ) were established as the lowest concentration that could amplify the 10 serial dilutions of gBlock used in the standard curve to determine the detection and quantification capacity of the proposed method, thus marking the minimum sensitivity genetic load for the assay. Analytical sensitivity was first determined using a synthetic gBlock, setting a limit of detection 1 copy/μL. To evaluate the performance of the assay in a biological matrix, the gBlock was tested on RNA extracted from jejunal tissue, confirming detection at the same limit despite the presence of host RNA.

### Analytical specificity of the RT–qPCR assay

To assess the specificity of the multiplex assay, RT–qPCR was performed via positive controls for other recurrent viruses in chickens: chicken parvovirus (ChPV), avian metapneumovirus subtypes A (AMPV-A) and B (AMPV-B), Newcastle disease virus (NDV) and fowl adenovirus 1 (FAdV-1), with ddH2O as the negative control.

### Analytical repeatability of the multiplex RT–qPCR assay

To assess repeatability, intra-assay and inter-assay tests were performed via serial dilutions of the synthetic DNA positive control to 10^7^ and 10^3^ DNA copies per μL, respectively, and the samples were subjected to the RT–qPCR protocol five times each over 5 days. Each of these serial dilutions was aliquoted separately and stored at −20°C until use. For inter-assay repeatability, an aliquot of each serial dilution was amplified by RT–qPCR five times under the same conditions. In each amplification, a different aliquot of serial dilution was thawed and used. For intra-assay repeatability, five serial dilutions of the gBlock described above were used, and 5 replicates were performed for each dilution factor. All procedures were performed according to MIQE (Real-Time PCR Experiment Standards) guidelines (67). A total of 5 trials were run using 5 different dilutions in quintuplicate on 5 different days. The aim of this procedure was to evaluate the stability of RT–qPCR. Cycle threshold (Ct) values and the calculated coefficient of variation (CV), as determined from the assay results, were used. Stability was calculated via the coefficient of variation for each corresponding point for the intra- and interassay evaluations.

### Sequencing and phylogenetic analysis

Randomly selected samples of each virus were subjected to RT-PCR amplification of a characteristic fragment. Portions of ORF1b as a partial method for characterization of ANV and CAstV and a part of the IBV S1 gene were amplified as previously described in 2007 and 1999 (49, 68). For ARV, a fragment of the σC gene was amplified, same with a fragment of the AvRVA NSP4 gene, as described previously in 2010 and 2007 (49, 69). All PCR protocols were performed via the Platinum™ Taq DNA Polymerase Kit (Invitrogen by Thermo Fisher Scientific, Carlsbad, CA, United States), with thermocycling conditions adjusted according to the melting temperature of each set of primers. The PCR products were subsequently visualized in a 2% agarose gel stained with SYBR Safe DNA Gel Stain (Invitrogen by Thermo Fisher ScientificCarlsbad, CA, United States). The amplified products were purified via an ExoSAP-IT™ Express PCR product Cleanup Kit (Applied Biosystems, Santa Clara, CA 95051, United States) following the manufacturer’s instructions. The purified products were then sequenced via the Big Dye Terminator v 3.1 cyclic sequencing kit (Applied Biosystems, Thermo Fisher Scientific) according to the manufacturer’s instructions on an ABI 3500 DNA analyzer (Applied Biosystems, Thermo Fisher Scientific). The sequences of each virus were aligned and analyzed alongside similar sequences randomly collected from GenBank from various regions worldwide. Each virus was analyzed separately via the CLUSTAL X 2.0.11 program (70). For each virus, a phylogenetic analysis was carried out via the neighbor–joining statistical method along with a p-distance substitution model and a bootstrap model phylogeny test with 1,000 repeats. This analysis was conducted via the MEGA version 7 software package (71), resulting in phylogenetic trees representing the characteristics of the sequences obtained for each virus.

### Statistical analysis

The virus detection data were organized through descriptive statistics and categorized by province, age range, and detection results for each virus. The data were then imported into the R Studio 2022.12.0 + 353 environment (72). First, a Shapiro–Wilk test was run to determine whether the virus detection data followed a normal distribution, a prerequisite for proceeding to multivariate testing. Upon confirmation, the chi-square test was applied to analyze the associations between categorical variables, such as province, age range and detection of each virus. A cutoff of *p* ≤ 0.05 was used to determine statistical significance.

The validation of the diagnostic method proposed in this study was carried out according to The MIQE guidelines and the WOAH Terrestrial Manual Chapter 1.01.06 and provide validation data for Stage 1 (67, 73). In order to determine the confidence in the diagnostic method, the calculation of the test performance metrics was applied using the following formulas: $\mathrm{Sensitivity}=\frac{TP}{TP+FN}$ , $\mathrm{Specificity}=\frac{TN}{TN+FP}$ , $\begin{array}{l} \mathrm{Positive}\;\\ \mathrm{predictive}\;\mathrm{value}\;\left(PPV\right)=\frac{\mathrm{Sensitivity}\;x\;\mathrm{prevalence}}{\begin{array}{l} \mathrm{Sensitivity}\;x\;\mathrm{prevalence}+\\ \left(1-\mathrm{Specificity}\right)\;x\;\left(1-\mathrm{prevalence}\right) \end{array}}\text{,} \end{array}$ $\begin{array}{l} \mathrm{Negative}\;\\ \mathrm{predictive}\;\mathrm{value}\;\left(NPV\right)=\frac{\mathrm{Specificity}\;x\;\left(1-\mathrm{prevalence}\right)}{\begin{array}{l} \mathrm{Specificity}\;x\;\left(1-\mathrm{prevalence}\right)+\\ \left(1-\mathrm{Sensitivity}\right)\;x\;\mathrm{prevalence} \end{array}}\text{,} \end{array}$ according to chapter 6 of Fletcher’s Clinical Epidemiology (74). Where: TP = true positives, FN = false negatives, TN = true negatives, FP = false positives, prevalence = frequency of enteric disease in chickens (11%), associated as the minimum probability of appearing to be enteric disease accompanied by malabsorption syndrome associated with viral pathogens (6, 75).

## Results

### Standard curves for single and multiplex RT–qPCR assays

**Figure 1:**
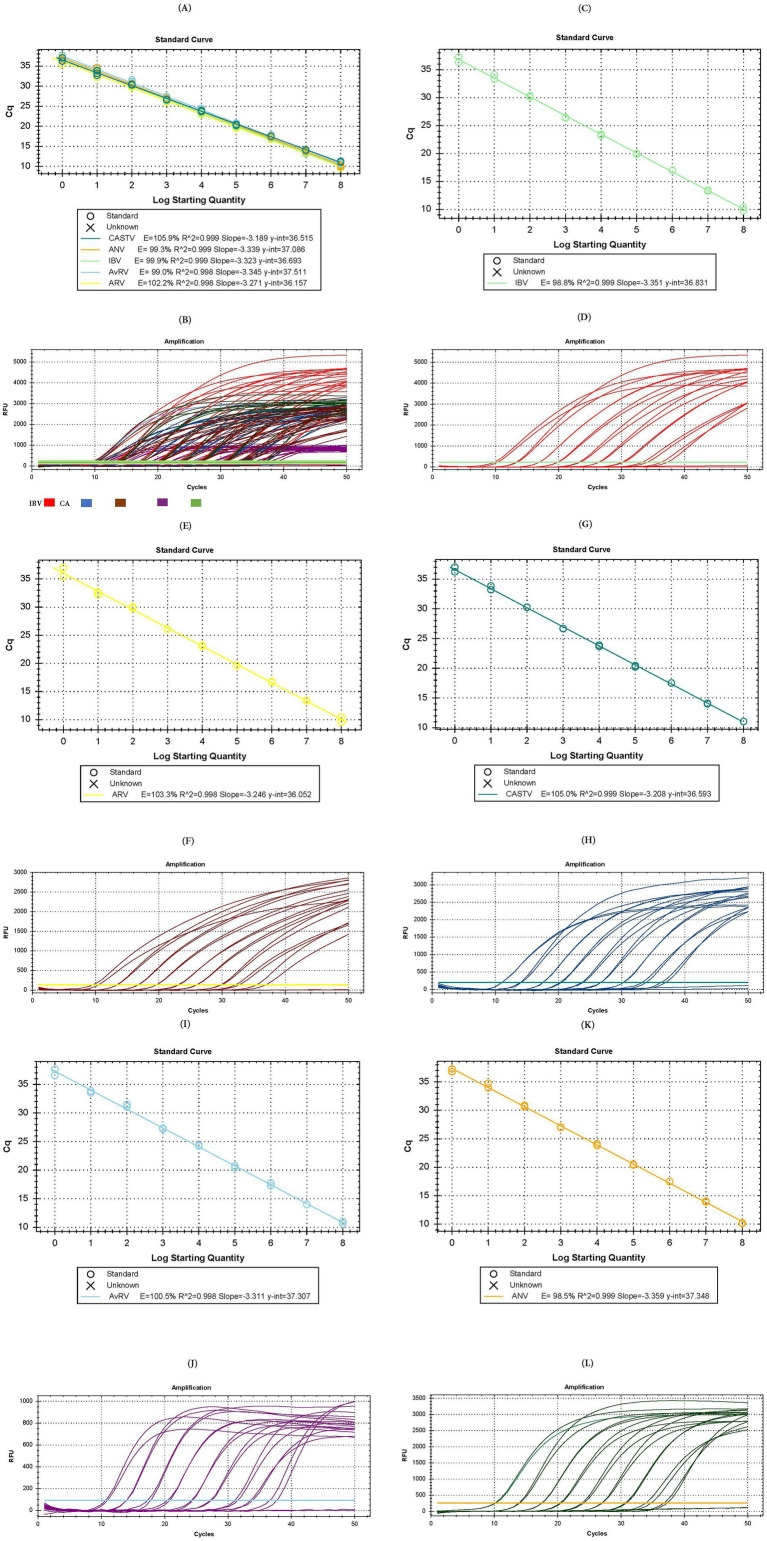
Test for the formulation of the standard curve in multiplex and single-plex assays. **(A)** Calibration curve of the multiplex assay. **(B)** Amplification plot of the multiplex assay [IBV in red color, ANV in green color, CAstV un blue color, AvRVA in purple color and ARV in brown color]. **(C)** Calibration curve of the single-plex assay for IBV. **(D)** Amplification plot of the single-plex assay for IBV. **(E)** Calibration curve of the single-plex assay for ARV. **(F)** Amplification plot of the single-plex assay for ARV. **(G)** Calibration curve of the single-plex assay for CAstV. **(H)** Amplification plot of the single-plex assay for CAstV. **(I)** Calibration curve of the single-plex assay for AvRVA. **(J)** Amplification plot of the single-plex assay for AvRVA. **(K)** Calibration curve of the single-plex assay for ANV. **(L)** Amplification plot of the single-plex assay for ANV.

To perform absolute quantification on the basis of the standard curve, assays were carried out to generate both multiplex and singleplex calibration curves. The multiplex calibration curve, using the 10 series dilutions of the synthetic DNA positive control, showed efficiencies of 105.9, 99.3, 99.9, 99.0 and 102.2% for CAstV, ANV, IBV, AvRVA and ARV, respectively, and correlation coefficients between 0.998 and 0.999 under previously established conditions (Figures 1A,B).

Curves generated in singleplex formats to examine their individual assay behavior demonstrated target amplification and efficiencies of 98.8% (Figures 1C,D), 103.3% (Figures 1E,F), 105.0% (Figures 1G,H), 100.5% (Figures 1I,J), and 98.5% (Figures 1K,L) for IBV, ARV, CAstV, AvRVA and ANV, respectively, and correlation coefficients between 0.99 and 1. Both the multiplex and singleplex assays exhibited an LoD and LoQ of up to 1 copy of genetic material per μL.

### Specificity of the RT–qPCR assay

The specificity of the RT–qPCR assay was confirmed by the absence of amplification from FAdV-1 and ChPV DNA; AMPV-A, AMPV-B and NDV RNA; and the non-template control (Supplementary material).

### Diagnostic test performance metrics

From the analysis carried out on the basis of the metrics of the diagnostic test proposed, a sensitivity of 97.03% was obtained, while the specificity was 100%. On the other hand, the NPV showed a result of 99.63% and the PPV of 100%, which indicates that the test has an excellent diagnostic capacity, both to affirm the presence of these pathogens and to indicate their absence.

### Repeatability of the assay

The intra-assay results for all the targets revealed high repeatability, where the most variable was 0.926% for CAstV, and the least variable was the ARV CV, which reached 0.248%. The intra-assay CVs were in accordance with the parameters, with the highest being 0.847% for IBV and the lowest being 0.425% for CAstV. Regarding the multiplex, the inter-assay results showed a coefficient of variability between 0.147 and 0.926%, and the intra-assay results showed a coefficient between 0.130 and 0.847%. This finding indicates that the multiplex and single-plex real-time RT–qPCR assay has low repeat variability (Table 2).

**Table 2:** Frequencies, Cq and St Dev of Inter- and Intraassay of multiplex RT-qPCR.

N°	Inter-Assay										Intra-Assay									
	CAstV	ANV	IBV	ARV	AvRVA	CAstV	ANV	IBV	ARV	AvRVA	CAstV	ANV	IBV	ARV	AvRVA	CAstV	ANV	IBV	ARV	AvRVA
	Cq* Mean					Cq St Dev					Cq Mean					Cq St Dev				
10^8	10.86	11.02	11.09	11.21	10.12	0.926	0.160	0.504	0.163	0.401	11.26	10.61	11.09	11.25	10.57	0.239	0.130	0.847	0.194	0.499
10^7	13.95	14.89	14.83	15.19	14.91	0.346	0.172	0.320	0.152	0.206	13.43	14.54	14.71	14.99	14.89	0.425	0.449	0.397	0.420	0.206
10^6	16.93	17.79	17.74	18.10	17.91	0.404	0.242	0.420	0.203	0.816	16.27	17.47	18.03	17.87	17.74	0.327	0.383	0.452	0.289	0.829
10^5	20.77	21.74	21.72	21.19	21.81	0.267	0.224	0.240	0.147	0.737	21.68	20.79	21.83	21.76	21.63	0.204	0.514	0.853	0.477	0.763
10^4	23.71	24.42	24.45	24.86	24.63	0.539	0.249	0.590	0.248	0.403	23.96	24.06	24.56	24.58	24.82	0.435	0.222	0.295	0.206	0.621

Cq, cycle of quantification.

### Enteric virus detection

Among the 200 chicken samples tested in this study, 97% (n = 194) were positive for at least one of the enteric viruses tested in the multiplex assay (Table 3). Among the 5 viruses detected, ANV was the most common, accounting for 89% of the positive samples (*p* value <0.001; Table 3). In contrast, AvRVA had the lowest presence, detected in only 11% of the samples from chickens with intestinal disease, showing no significant difference from the other groups (*p* value >0.05). In the Broiler group, all samples from birds aged 132 days were found to be infected by at least one of the 5 viruses tested in this study without showing significant differences from the remaining group (over 32 days of age). In both the Layer and Breeder groups, at least one of the viruses was found to be present in all birds up to 30 weeks of age, with no significant differences between the remaining groups (Table 3).

**Table 3:** Results of multiplex RT–qPCR assay detection for the five RNA viruses tested in this study.

Multiplex detection of enteric viruses in chickens									
Group	Age		N°. samples	CAstV	ANV	IBV	ARV	AvRVA	Total of positive samples (%)
				N°(+)	N°(+)	N°(+)	N°(+)	N°(+)	
Broiler	Days	1–7	16	8 (50%)	14 (87%)	1 (6.25%)	1 (6.25%)	1 (6.25%)	16 (100%)
		8–14	13	7 (53.9%)	9 (69.2%)	6 (46.2%)	2 (15.4%)	1 (7.7%)	13 (100%)
		15–32	25	14 (56%)	23 (92%)	6 (24%)	5 (20%)	3 (12%)	25 (100%)
		>32	68	31 (45.6%)	64 (94.1%)	9 (13.2%)	4 (5.9%)	5 (7.4%)	65 (95.6%)
Layer	Weeks	1–30	37	21 (56.8%)	31 (83.8%)	15 (40.5%)	7 (18.9%)	5 (13.5%)	37 (100%)
		>30	35	21 (60%)	31 (88.6%)	9 (25.7%)	6 (17.1%)	7 (20%)	32 (91.4)
Breeder		1–30	5	3 (60%)	5 (100%)	3 (60%)	0 (0%)	0 (0%)	5 (100%)
		>30	1	1 (100%)	1 (100%)	1 (100%)	0 (0%)	0 (0%)	1 (100%)
Total			200	106 (53%)	178 (89%)*	50 (25%)	25 (12.5%)	22 (11%)	194 (97%)

*Significant differences according to statistical analysis compared with other parameters.

Regarding viral load, broilers presented the highest average viral load for CAstV, ANV and IBV and in layers for ARV (Table 4), with significant differences from the layer and breeder groups for CAstV (*p* value <0.001) and no differences for the remaining 4 viruses (*p* value >0.05). In particular, AvRVA presented the highest average viral load, with no significant differences from the other viruses, whereas ARV presented the lowest average viral load (Table 4).

**Table 4:** Quantification of the five RNA viruses tested in this study via multiplex RT–qPCR.

Quantification of enteric viruses in chickens												
Group	Age		CAstV		ANV		IBV		ARV		AvRVA	
			Av. GC	Mx. GC	Av. GC	Mx. GC	Av. GC	Mx. GC	Av. GC	Mx. GC	Av. GC	Mx. GC
Broiler	Days	1–7	15	204	3,919	52,821	13	13	15	15	4,412	4,412
		8–14	29,001	375,909	385,924	4,179,027	204,973	2,288,837	4	33	2	32
		15–32	886,256	8,265,264	2,482,739	47,461,888	53,953	704,740	50,985	1,273,578	92	2,268
		>32	433,904	28,768,860	18,307	268,453	3,339	171,445	914	32,365	14	961
Layer	Weeks	1–30	237	3,596	386,129	14,169,410	14,962	391,053	4	40	23	494
		>30	97,677	2,215,496	174,560	2,665,836	97,821	1,782,251	84,178	2,945,468	13,277,023	464,695,237
Breeder		1–30	1	2	167,581	837,855	31,017	133,951	N/A	N/A	N/A	N/A
		>30	11	11	3,126	3,126	2	2	N/A	N/A	N/A	N/A
Total			277,333	28,768,860	448,243	47,461,888	42,151	2,288,837	21,421	2,945,468	2,323,522	464,695,237

Av, Average; Mx, Maximum; GC, Gene copies per μL; N/A, Not applicable.

**Figure 2:**
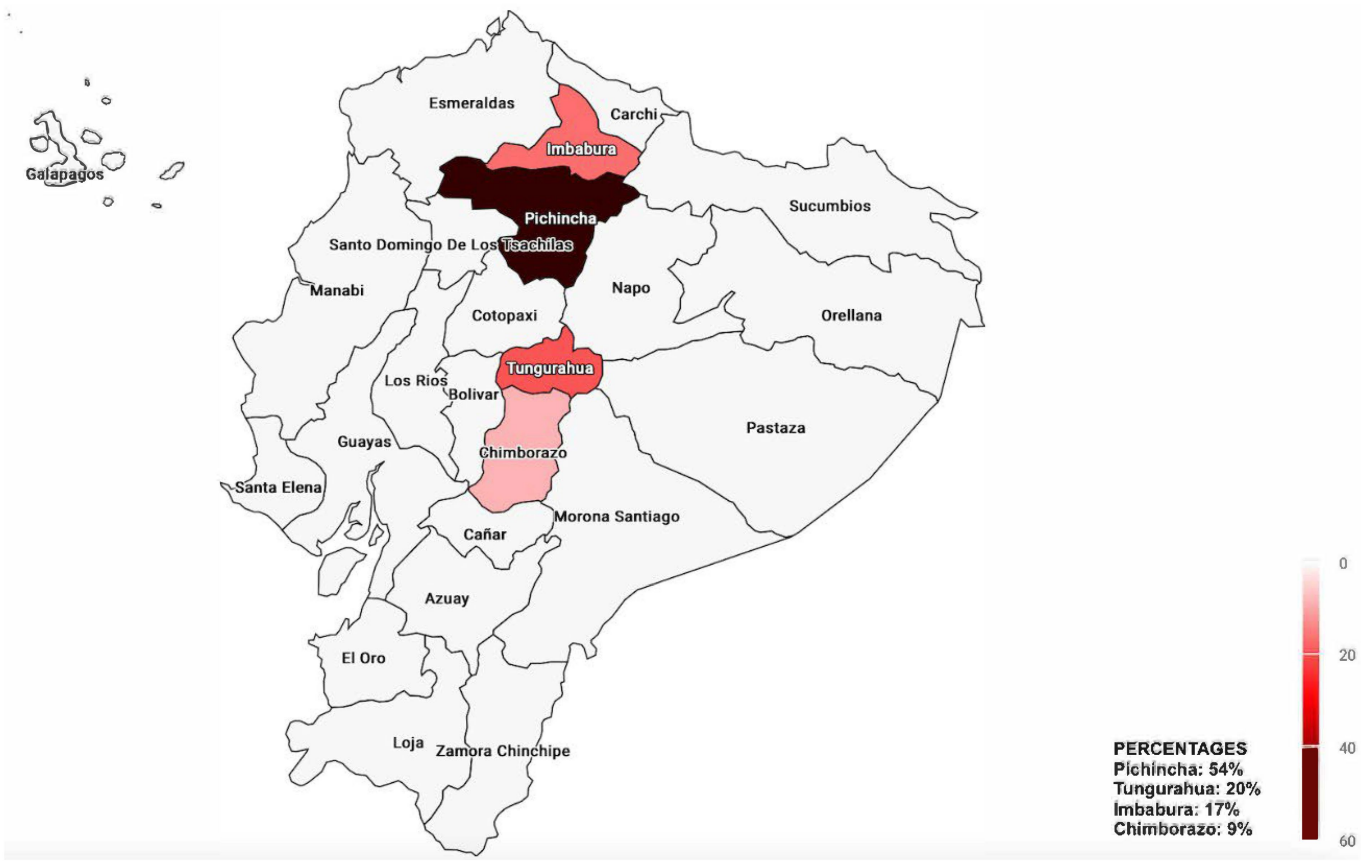
Map of the percentage of positive samples for each virus across the provinces of Ecuador. Provinces not sampled are shown in gray.

When virus detection in relation to sample origin was analyzed, Pichincha was the province with the highest percentage of positive samples for each virus analyzed, although the difference was statistically significant only for IBV (*p* value = 0.0187; Table 5). Conversely, Chimborazo showed the lowest percentage of positivity for all viruses tested. Across all provinces, ANV and CAstV were the viruses with the highest positivity rates, regardless of the number of samples per province (Figure 2). However, these data are limited by the unequal number of samples for each province and the overall study.

**Table 5:** Results of the multiplex RT–qPCR assay for the five tested RNA viruses in relation to the locality of the samples.

Provinces	CAstV	ANV	IBV	ARV	AvRVA
	Number of positive samples/Number of total samples (%)				
Chimborazo	10/24 (5.0%)	22/24 (11.0%)	2/24 (1.0%)	1/24 (0.50%)	1/24 (0.50%)
				
Imbabura	17/32 (8.5%)	26/32 (13.0%)	13/32 (6.5%)	4/32 (2.0%)	3/32 (1.50%)
				
Pichincha	58/112 (29.0%)	102/112 (51.0%)	24/112 (12.0%)*	12/112 (6.0%)	11/112 (5.50%)
				
Tungurahua	21/32 (10.5%)	28/32 (14.0%)	11/32 (5.5%)	8/32 (4.0%)	7/32 (3.50%)
				

*Significant differences between every enteric virus present in the analyzed provinces according to the chi-square test.

### Coinfection analysis

Among the 194 positive samples, 124 (63.9%) presented coinfections with up to five different viruses (Table 6). Among the 31 possible combinations of the five viruses, 18 different combinations were detected. Interestingly, there were no single infections of ARV; this virus was observed only in coinfections with other viruses. Three samples tested positive for all five viruses simultaneously (Table 6, combination C1). Coinfection with CAstV and ANV appeared to be the most frequent combination, detected in 62 samples. However, no significant difference was detected between the frequencies of the different combinations (*p* value>0.05). Single infections with CAstV, IBV and AvRVA were reported in 6, 2 and 2 samples, respectively, while 60 samples tested positive for ANV alone. Therefore, in chickens with intestinal disease, ANV was the most frequently detected virus, both in single infections and coinfections.

**Table 6:** Combinations of the viruses detected in this study.

Coinfections	N°C	CAstV	ANV	IBV	ARV	AvRVA	N° (+)(%)
5 viruses	C1	x	x	x	x	x	3 (1.55%)
4 viruses	C2		x	x	x	x	3 (1.55%)
	C3	x		x	x	x	2 (1.03%)
	C4	x	x		x	x	2 (1.03%)
	C5	x	x	x		x	0 (0.00%)
	C6	x	x	x	x		7 (2.61%)
3 viruses	C7	x	x	x			20 (10.31%)
	C8		x	x	x		0 (0.00%)
	C9			x	x	x	0 (0.00%)
	C10		x		x	x	2 (1.03%)
	C11	x			x	x	0 (0.00%)
	C12	x		x	x		0 (0.00%)
	C13		x	x		x	0 (0.00%)
	C14	x		x		x	0 (0.00%)
	C15	x	x		x		2 (1.03%)
	C16	x	x			x	2 (1.03%)
2 viruses	C17	x	x				62 (31.96%)
	C18	x		x			0 (0.00%)
	C19	x			x		0 (0.00%)
	C20	x				x	0 (0.00%)
	C21		x	x			12 (6.19%)
	C22		x		x		0 (0.00%)
	C23		x			x	3 (1.55%)
	C24			x	x		1 (0.52%)
	C25			x		x	0 (0.00%)
	C26				x	x	3 (1.55%)
Single	C27	x					6 (3.09%)
	C28		x				60 (30.93%)
	C29			x			2 (1.03%)
	C30				x		0 (0.00%)
	C31					x	2 (1.03%)

N°C, Combination number. C1-31 = −31. N°(+) = Number of positive samples.

### Sequencing and phylogenetic analysis

#### CastV

**Figure 3:**
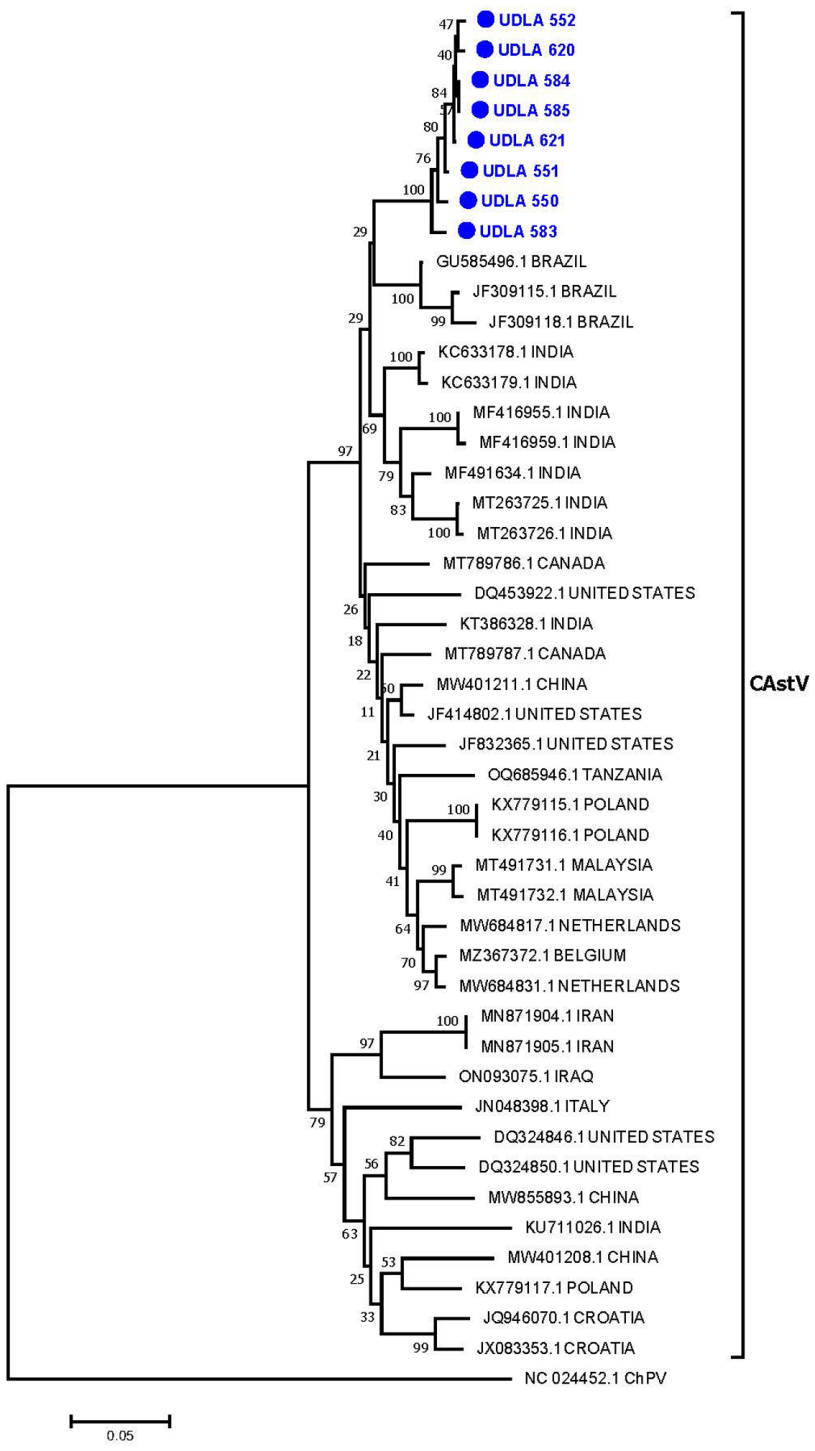
Phylogenetic relationships between the sequences of CAstV obtained here and other sequences of CAstV from Brazil, India, Canada, the United States, China, Tanzania, Poland, Malaysia, the Netherlands, Belgium, Iran, Iraq, Italy, and Croatia based on part of the ORF 1b gene nucleotide sequence. Sequences were aligned via the CLUSTAL W method in ClustalX2 2,1. The phylogenetic tree was constructed via the MEGA 7 software package. Numbers along the branches refer to bootstrap values for 1,000 replicates. The scale bar represents the number of substitutions per site. Chicken parvovirus (ChPV) was used as the outgroup. Sequences obtained in this study are in blue and marked with ●.

Phylogenetic analysis on the basis of randomly selected CastV ORF1b sequences generated a tree that grouped the sequences into two main clades and several subclades. Our Ecuadorian sequences formed a distinct cluster (bootstrap 100%) closely related to previously published sequences from Brazil (Figure 3). The 8 sequences obtained in this study presented 97.5 to 99.7% nucleotide (NT) similarity among themselves and 90.86 to 93.91% similarity with the Brazilian sequences (Supplementary material). Additionally, these sequences presented approximately 90% NT similarity with sequences from Belgium, Canada, China, India, Malaysia and the United States and approximately 80% NT similarity with previously published sequences from Poland, Iran, Italy, Iraq, Croatia, and the Netherlands.

#### ANV

**Figure 4:**
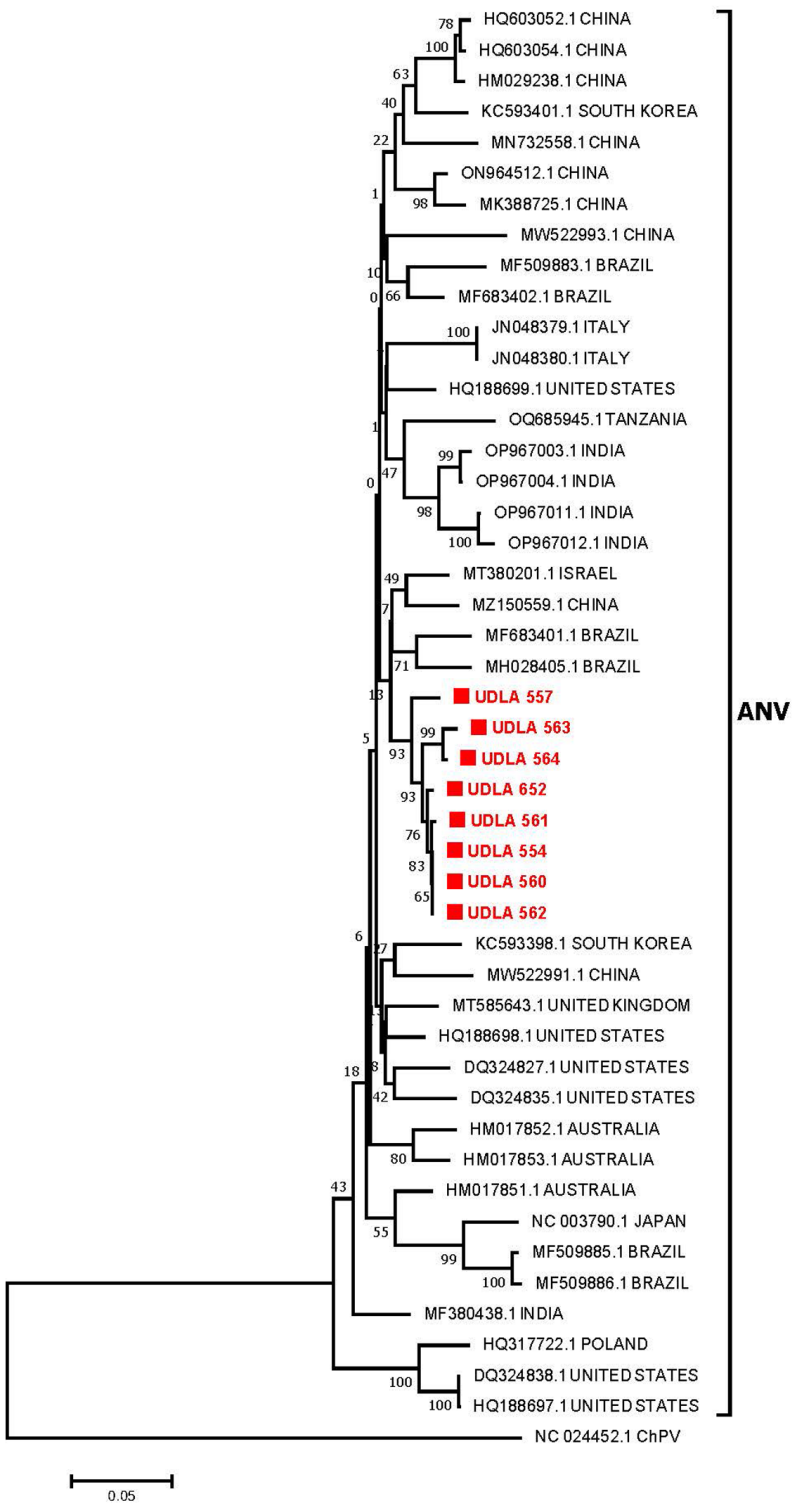
Phylogenetic relationships between the ANV sequences obtained here and other ANV sequences from China, South Korea, Brazil, Italy, the United States, Tanzania, India, Israel, the United Kingdom, Australia, Japan, and Poland based on a portion of the ORF 1b gene nucleotide sequence. Sequences were aligned via the CLUSTAL W method in ClustalX2 2,1. The phylogenetic tree was constructed via the MEGA 7 software package. Numbers along the branches refer to bootstrap values for 1,000 replicates. The scale bar represents the number of substitutions per site. Chicken parvovirus (ChPV) was used as the outgroup. Sequences obtained in this study are in red and marked with ■.

The phylogenetic analysis of the partial ANV ORF1b sequences revealed that the sequences obtained in this study were grouped into a single clade (93% bootstrap). These sequences were found to be closer to two Brazilian sequences (MH028405 and MF683401) and were grouped in the same clade as sequences from China and Israel (Figure 4). The sequences from this study presented 97.46 to 100% NT similarity. Furthermore, they presented between 86 and 97% NT similarity with sequences from Brazil, Israel, and China; between 89 and 95% NT similarity with sequences from Australia, Italy, South Korea, Tanzania, and the United States; and approximately 87% NT similarity with sequences from Japan and other Brazilian sequences (Supplementary material).

#### IBV

**Figure 5:**
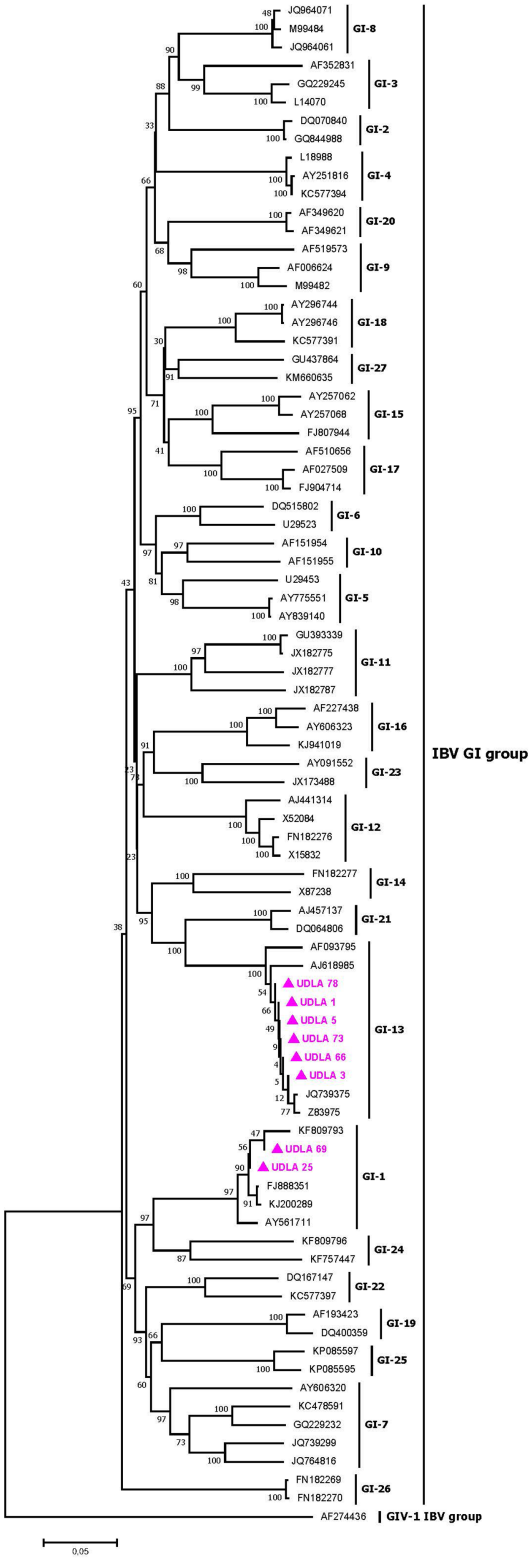
Phylogenetic relationships between the sequences of IBV obtained here and other sequences of IBV belonging to the GI groups of IBV classification based on part of the S1 gene nucleotide sequence. Sequences were aligned via the CLUSTAL W method in ClustalX2 2,1. The phylogenetic tree was constructed via the MEGA 7 software package. Numbers along the branches refer to bootstrap values for 1,000 replicates. The scale bar represents the number of substitutions per site. Sequences obtained in this study are pink and marked with ▲.

The phylogenetic analysis of IBV partial S1 gene sequences from the GI group revealed different GI lineages, ranging from GI-1 to GI-27, as previously described by Valastro et al. (31). The IBV sequences obtained in this study were grouped into two distinct lineages: six sequences grouped in the GI-13 clade, along with European strains such as the live attenuated vaccine strains 793B and 4/91, and two sequences grouped in the GI-1 cluster, along with North American strains, particularly the Massachusetts vaccine strain (Figure 5). Among the six sequences in the GI-13 group, all sequences presented 100% NT similarity with the strain sequence JQ739375 (Supplementary material) which has a minimal difference with the vaccine strain. The two sequences in the GI-I group presented 100 and 98% NT similarity to the Massachusetts vaccine strain, corresponding to a vaccine strain and a wild-type strain, respectively (Supplementary material) (36; Figure 5).

#### ARV

**Figure 6:**
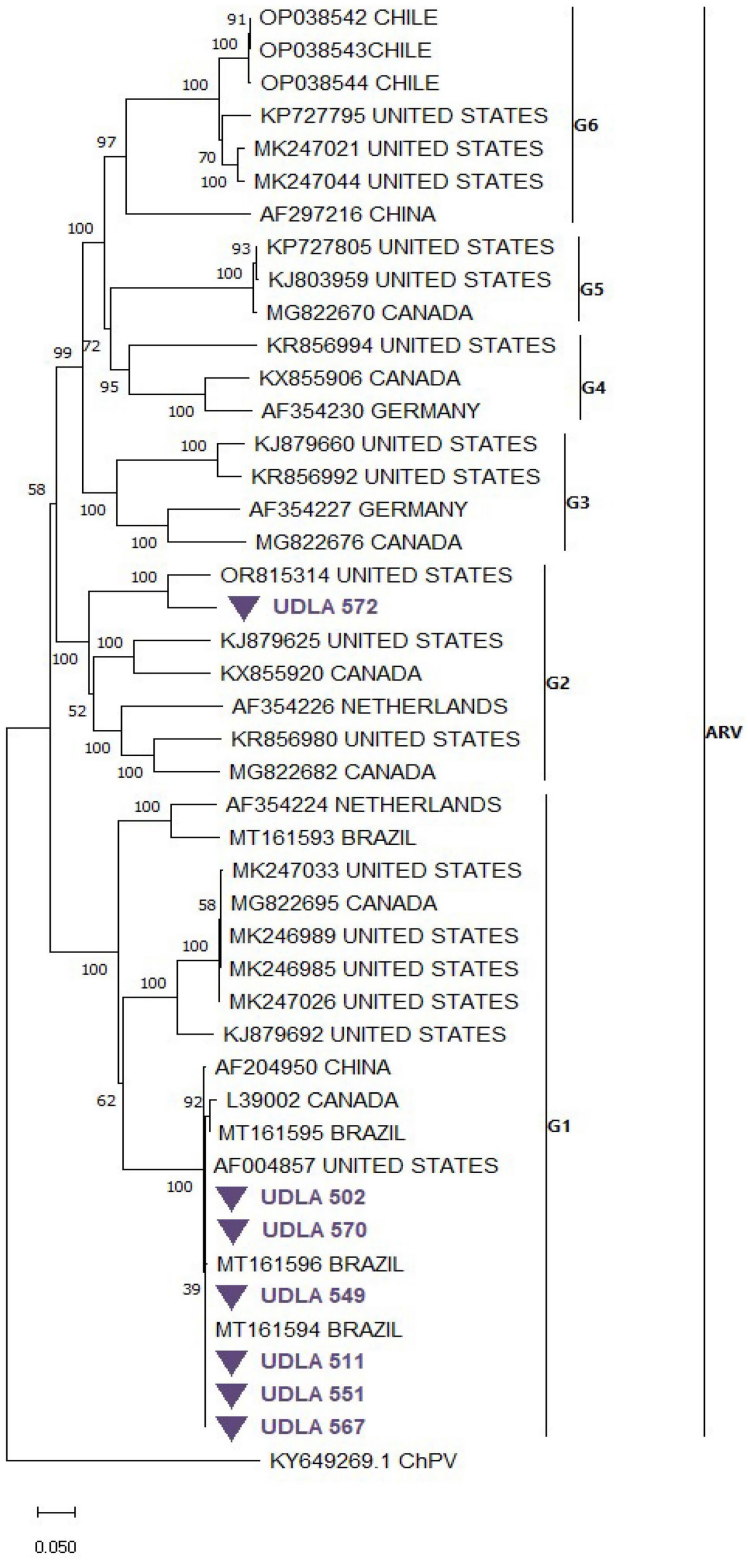
Phylogenetic relationships between the sequences of ARV obtained here and other sequences of ARV belonging to genotypes (G) of ARV based on part of the S1 nucleotide sequence coding to the σC protein. Sequences were aligned via the CLUSTAL W method in ClustalX2 2,1. The phylogenetic tree was constructed via the MEGA 7 software package. Numbers along the branches refer to bootstrap values for 1,000 replicates. The scale bar represents the number of substitutions per site. Sequences obtained in this study are purple and marked with ▼.

The phylogenetic analysis of ARV S1 segment sequences, which were randomly collected from GenBank along with those obtained in this study, resulted in a tree with distinct clades for genotypes 1 to 6 (Figure 6). Six of our sequences (UDLA 502, 570, 549, 511, 551 and 567) grouped within the genotype 1 cluster, closely related to previously published sequences from Brazil and the United States. The remaining sequence (UDLA 572) was assigned to the genotype 2 clade and showed proximity to sequences from the United States. When the NT similarities between these sequences were analyzed, those assigned to genotype 1 presented 98–99% NT similarity with the Brazilian and Unites States sequences, with the similarity decreasing to 70% with other sequences within genotype 1. Furthermore, the UDLA 572 sequence had only 87–90% NT similarity with the closest U.S. sequence (OR815314) and as low as 70% similarity with other sequences from genotype 2 (Supplementary material; Figure 6).

**Figure 7:**
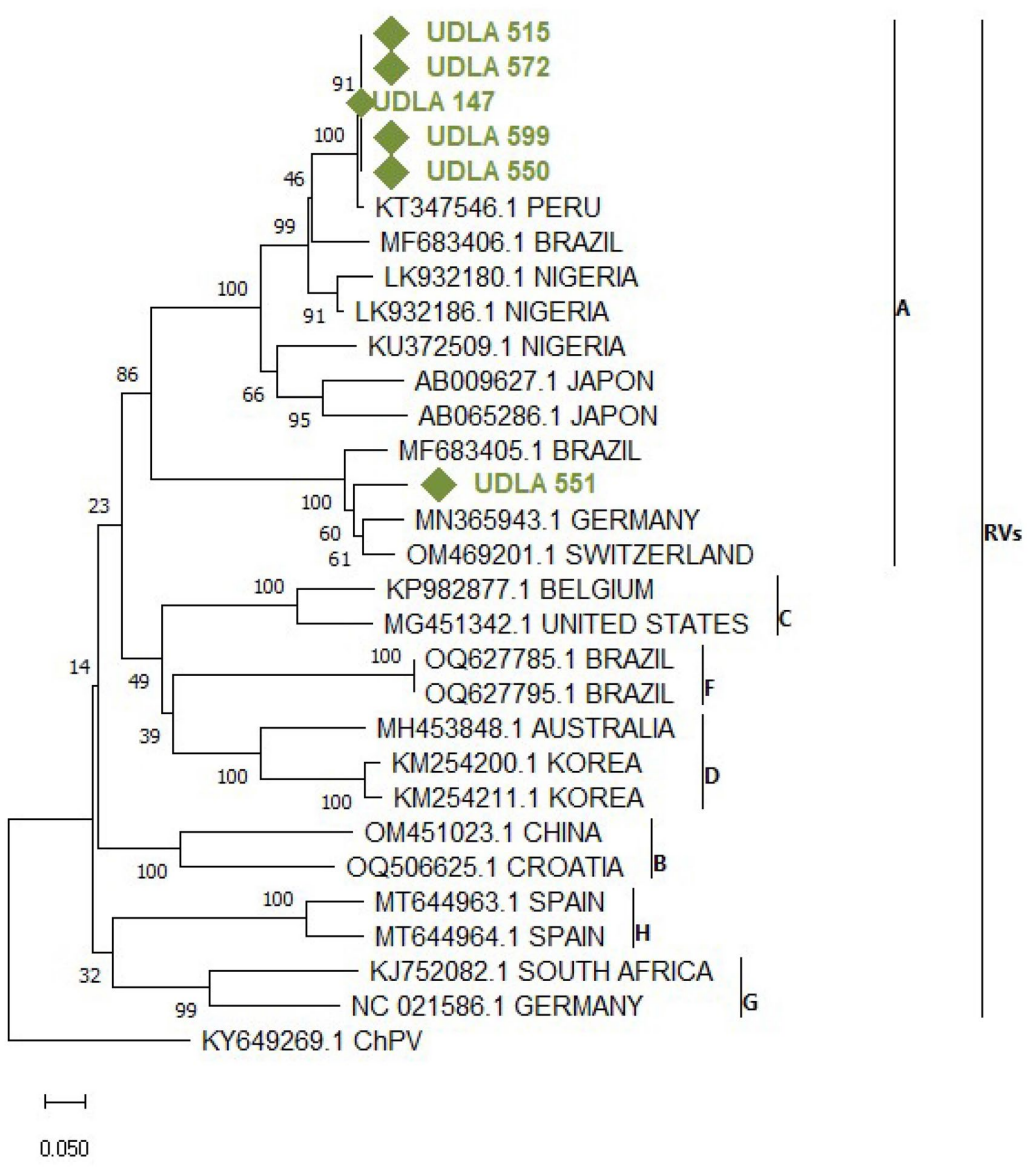
Phylogenetic relationships between the sequences of AvRV obtained here and other sequences of AvRV belonging to Groups A-H classification on the basis of a fragment of NSP4 gene nucleotide sequences. Sequences were aligned via the CLUSTAL OMEGA method in ClustalX2 2,1. The phylogenetic tree was constructed via the MEGA 11 software package. Numbers along the branches refer to bootstrap values for 1,000 replicates. The scale bar represents the number of substitutions per site. Sequences obtained in this study are in green and marked with ♦.

#### AvRVA

Through phylogenetic analysis of AvRV NSP4 gene sequences from both GenBank and our study, a phylogenetic tree delineating separate clades corresponding to Groups A to H was generated. As expected, all sequences obtained in this study fell within Group A. Specifically, the sequence UDLA 551 was grouped with sequences from Brazil, Germany and Switzerland (Figure 7), exhibiting 88–91% NT similarity with them. Conversely, the remaining sequences (UDLA 515, 572, 147, 599 and 500) were more closely related to previously published sequences from Peru and Brazil, displaying 88–97% NT similarity with them and decreasing to 37% similarity with UDLA 551 (Supplementary material).

## Discussion

This study analyzed the presence of CAstV, ANV, IBV, AvRVA and ARV in Ecuadorian poultry with enteritis. A novel multiplex RT–qPCR assay was developed and standardized for the detection of these five enteric viruses. The inclusion criterion was the use of sequenced and genotypically characterized strains. The exclusion criterion involved genomes of other enteric and respiratory viruses detected in poultry, to prevent potential cross-reactivity. Highly conserved genomic regions were selected for primer and hydrolysis probe design and compared against all available sequences in GenBank for each target virus to ensure both broad detection capacity and specificity. Although there are no commercial vaccines for most of the viruses included in the assay, sequences from vaccine strains of ARV (1,133, 2,408) and IBV (GI-1, GI-13) were included in the in silico analysis to confirm specificity. Assay exclusivity was further validated against genomes of non-target viruses such as ChPV, AMPV-A, AMPV-B, NDV, and FAdV-1, with no cross-hybridization detected. Sensitivity, defined by the limit of detection and quantification for each RT-qPCR target, confirmed the assay’s high analytical performance. It also exhibited high sensitivity by being able to amplify even a single copy of viral material (Figure 1) and high repeatability, with variation coefficients of less than 10% for interassay and intra-assay methods (Table 2), meeting established parameters for molecular diagnostic trials (56, 76).

The astroviruses ANV and CAstV were the most commonly detected viruses in chickens with intestinal disease in our study, with notably high detection rates of 89 and 53%, respectively. These findings are particularly concerning, as these viruses are linked to diseases such as RSS, kidney disease, visceral gout and, in certain strains, white chicken syndrome (20, 24, 77, 78). The high prevalence of these viruses in breeder hens is especially alarming because they can be transmitted vertically to progeny during embryonic development (79). This poses significant risks to chicken breeding, given that certain genotypes of CAstV and ANV can cause mortality rates of up to 60% in the first days after birth. Additionally, white chicken syndrome, caused by the CAstV genotype B *iv*, leads to 100% mortality within a few days of hatching (80, 81). Despite its recent report in the country (27), ANV has been the most prevalent virus in the samples analyzed. The lack of vaccines to combat infections related to this virus heightens the emerging risk in poultry.

This study conducted phylogenetic analysis of ANV and CAstV on the basis of the coding sequence of the RdRp gene, as it is easily amplified and sequenced (49, 82). However, since this gene is the most conserved region of the astrovirus genome, it was not possible to perform genotyping according to the previously described genotypes, ANV 1 and 2, and CAstV A and B. Therefore, future studies should focus on sequencing the ORF2 gene, which encodes the capsid protein and enables genotyping (83–85), which would facilitate the identification of the circulating strains and genotypes within the country, along with their associated pathogenicity and virulence, ultimately informing the development of appropriate measures to address the impact of this disease. Nevertheless, for both viruses, the sequences showed greater proximity and percentage of NT similarity with strains previously found in Brazil (Figures 3, 4; Supplementary material). Importantly, previous reports from Brazil have identified the two genotypes of ANV and the genotypes A and B of CAstV, including those associated with RSS and white chicken syndrome (6, 81). These findings present emerging risks that poultry farmers in the country need to consider.

After astroviruses, the most common virus detected in this study was IBV (Table 3). This virus is particularly concerning not only because it causes enteric symptoms but also because it is well known for causing severe respiratory disease (86). Therefore, its persistent prevalence in poultry poses significant risks for chicken breeding. Layers and breeder hens presented the highest frequencies of IBV infection, which is especially problematic since this virus is associated with eggshell deformities and alterations in egg white chemistry, leading to lower product quality (32, 68).

Our results demonstrated the circulation of strains from the IBV GI-1 and GI-13 groups on poultry farms in the country. For the identified sequences of the two genotypes in this study, strains classified as wild according to their percentage of variation were found in comparison with vaccine strains, given that vaccination for these two genotypes is carried out in Ecuador (33, 36). The circulation of wild strains of this virus in birds of all ages suggests a potential decrease in the efficacy of vaccination against these strains (87). In Ecuador, the circulation of wild GI-16 strains has been previously reported (37), but none of these studies were based on the complete sequence of the S1 region. Similarly, the presence of strains that have minimal differences to commercial vaccines may be due to mutations of the vaccine strain (88, 89), named vaccine-derived strains, which may have undergone a reversal of inactivation and reactivation of the pathogenic effect of the strain, however, as the complete sequence of the S1 region is not yet known (31), it is not possible to identify this dangerous phenomenon. Complete sequencing of the S1 region has been established as the most reliable method for differentiating IBV strains (29, 31). Coinfection of different strains in the same individual can increase virulence, making it crucial for future studies in the country to analyze and identify all circulating strains by S1 complete sequencing at a national level and determine whether there are unique or derived strains that affect virus behavior.

ARV was detected in only the broiler group (Table 3), and its pathogenic characteristics suggest that poultry risks should be considered. All samples positive for ARV were coinfected with other viruses, which may be attributed to the immunodepressive effects associated with ARV (56, 90), leading to increased susceptibility to infections with other enteric viruses in this study. Tenosynovitis, or viral arthritis, is the main differential pathology of avian reoviruses (39, 91) and is characterized by inflammation in tendons and joints. In birds intended for consumption, these symptoms result in lameness, depression and up to a 20% reduction in growth among infected birds (39), which could cause a decrease in poultry production; therefore, these birds are unable to meet the demand for fattening due to these infections. Among the sequences analyzed, which were based on the coding sequence for the σC protein (92), genotypes 1 and 2 were identified (Figure 6). Previous studies have analyzed the ability of commercial vaccines to combat infection by wild-type ARV strains and reported that, for both genotype 1 and genotype 2 (93, 94), vaccines based on strains close to genotype 2 to prevent Viral Tenosynovitis are not able to offer sufficient protection for strains causing RSS or enteric diseases of genotype 1. In particular, wild-type strains of genotype 1 show a greater ability to survive the effects of acquired immunity caused by commercial vaccines (39, 95), which is related to the fact that most of the samples that were sequenced corresponded to this genotype and are predominant in enteric infections.

Although AvRVA presented the lowest number of positive samples among the pathogens studied, a single sample presented the highest viral load among all the samples and viruses tested (Tables 3, 4). This observation may be attributed to the circulation of hypervirulent strains recently identified as emerging in poultry populations worldwide (96). These strains have demonstrated the ability to evade vaccine immunity and exhibit a relatively high incidence of feed malabsorption. However, to conduct a virulence analysis, data on the VP6 protein are needed (47, 97). Therefore, it is necessary to study the sequences encoding this protein in strains circulating in the country in the future.

Additionally, the quantification carried out in this study allows monitoring of the viral load of each virus in relation to the occurrence of RSS in poultry, since all the viruses tested are linked to the occurrence of this disease (24, 25, 90, 96, 98). In the age-specific analysis (Table 4), the highest viral load for each virus was found in the chicken groups older than 7 days of age. This finding has been linked to the occurrence of the most severe enteric disease effects observed in chickens where the virus was transmitted horizontally, leading to increased mortality rates of up to 40% for viruses such as IBV (86). Since evidence of enteric diseases is most evident from 10 days of age according to previous reports, monitoring the causative agent or multiple causes via the viral load test proposed in this study would generate future specialized diagnoses for each unique case in poultry with this condition.

All 5 viruses analyzed are transmitted horizontally, and transmission of these viruses between sick and healthy chickens represents an increasing risk in each of the seropositive groups, exponentially increasing the occurrence of enteric diseases. Therefore, despite the low number of positive samples for ARV and AvRVA, these viruses could start a cascade of contagion in poultry farms, generating problems in the future given the symptomatology of these viruses. By simultaneously identifying and quantifying these 5 enteric viruses in chickens with enteritis, this study enables the detection of potential coinfections that may exacerbate issues in infected poultry (Table 6). Previous reports have indicated that coinfections with multiple types of astroviruses in the same individual can increase the virulence of the strains and increase the mortality rate in young birds (28, 99). Since the combination of CAstV and ANV is the most common combination in the samples tested, it implies high risks for poultry production, especially in breeding hens (100). The combinations involving these astroviruses and IBV were predominant over the others (Table 6); the addition of this third infectious agent amplifies the potential adverse effects on the birds by introducing respiratory disease into the clinical picture, which is traditionally associated with IBV, as well as uterine damage detrimental to egg formation, which is crucial to the performance of layers and breeders (68, 101, 102). For young birds, this could lead to a lethal scenario. Finally, several samples in this study presented both 4 and 5 viruses. This could be due to increased susceptibility to other infections in the presence of one virus, leading to an infectious cascade culminating in the presence of all these pathogens. Additionally, this leads to increased intestinal symptoms and mortality (9, 103, 104). Therefore, in the future, identifying the concurrent pathologies associated with the presence of multiple viruses in a single patient is necessary.

Although intestinal disease in poultry involves nonviral factors, such as bacteria (105), parasites (106) and feed imbalance (107), viruses stand out as the main cause of these disorders (5, 30, 108). Accordingly, our study revealed an infection rate of 97% for any of the 5 studied enteric viruses in chickens suffering from enteritis in Ecuador. Early diagnosis is essential to effectively manage intestinal disorders in poultry, enabling proactive disease control and resolution. However, it is crucial to implement biosecurity protocols to regulate infections, such as reducing contact with germs through proper biosecurity practices and effective stress mitigation. This study serves as an approach for developing an efficient diagnostic method for the most common enteric viruses and analyzing their presence in Ecuador. This is the first RT-qPCR-based diagnostic method for these enteric viruses associated mainly with economic losses in poultry, so its commercial application could positively influence the epidemiological control of these viruses. Therefore, to continue monitoring these viruses, subsequent studies are necessary to investigate the pathological impact of these viruses in the country and the unique strains that may circulate in poultry farms within this territory.

## Conclusion

This study developed and validated a novel multiplex RT-qPCR assay to detect five key enteric viruses in Ecuadorian poultry: CAstV, ANV, IBV, AvRVA, and ARV. The assay exhibited high specificity, sensitivity, and repeatability, making it a valuable tool for diagnosing viral enteritis. Notably, ANV and CAstV were the most prevalent, posing significant risks due to their high virulence, association with severe syndromes like RSS, and vertical transmission capabilities. IBV strains of wild genotypes GI-1 and GI-13, as well as ARV and AvRVA, demonstrated potential to undermine vaccination efforts and cause economic losses due to decreased production. Coinfections, particularly involving astroviruses and IBV, amplified disease severity, highlighting the need for comprehensive viral monitoring and genomic studies. The study underscores the importance of sequencing specific genomic regions (e.g., ORF2 for astroviruses and S1 for IBV) for genotyping and understanding viral evolution. Future research should prioritize these methods to refine epidemiological surveillance, enhance vaccination strategies, and mitigate the impact of enteric diseases in poultry. To conclude with the validation of the method proposed here, and to promote its commercial and research use for the diagnosis of these viruses, it is necessary to include samples from different geographical areas with different prevalences to see if it can detect them in latent models.

## Data Availability

The nucleotide sequences of a portion of sequenced gene fragments identified in the present study for each virus were deposited in GenBank under the following accession numbers. For CAstV sequences: PQ586387 (UDLA 552), PQ586388 (UDLA 620), PQ586389 (UDLA 584), PQ586390 (UDLA 585), PQ586391 (UDLA 621), PQ586392 (UDLA 551), PQ586393 (UDLA 550), PQ586394 (UDLA 553). For ANV sequences: PQ586395 (UDLA 557), PQ586396 (UDLA 563), PQ586397 (UDLA 564), PQ586398 (UDLA 652), PQ586399 (UDLA 561), PQ586400 (UDLA 554), PQ586401 (UDLA 560), PQ586402 (UDLA 562). For IBV sequences: PQ586403 (UDLA 78), PQ586404 (UDLA 1), PQ586405 (UDLA 5), PQ586406 (UDLA 73), PQ586407 (UDLA 66), PQ586408 (UDLA 3), PQ586409 (UDLA 69), PQ586410 (UDLA 25). For ARV sequences: PQ586380 (UDLA 572), PQ586381 (UDLA 502), PQ586382 (UDLA 570), PQ586383 (UDLA 549), PQ586384 (UDLA 511), PQ586385 (UDLA 551), PQ586386 (UDLA 567). For AvRVA sequences: PQ586374 (UDLA 515), PQ586375 (UDLA 572), PQ586376 (UDLA 147), PQ586377 (UDLA 599), PQ586378 (UDLA 550), PQ586379 (UDLA 551).
