# Supplementary material for: Simultaneous detection and partial molecular characterization of five RNA viruses associated with enteric disease in chickens: chicken astrovirus, avian nephritis virus, infectious bronchitis virus, avian rotavirus a and avian orthoreovirus, via multiplex RT–qPCR
Source: Front Vet Sci. 2025 Apr 24;12:1536420. doi: 10.3389/fvets.2025.1536420 (PMC12058722; doi:10.3389/fvets.2025.1536420)
Supplement: Supplementary file 1 [file Data_Sheet_1.PDF]

Supplementary Material

1.1 Supplementary material: GBlock Sequence

CCCATGAGACATACAAAAAGGTAATGCCGCCAGTTACTACACCCCAGGGGCAACGTTGA
TGCTCCTAAAAAACTCTGGCTGGACGCAAGCCGTAACACCCGTGTCACTTCATAATGAG
AATGCTGATCCCGACGTTAGGGACTACTTAGATAAGCAAATTCACTGCGTCGAGGAGTA
TGCTGCTGCTGAAGATATACAGTTACCAGAAGTCGGGCCCGACTTCTTTCAGAAAATCT
GGTAGAGGGATGGACCGAAATAGAGCAGCATGGCCGATAAGGCTGGGCCGCAGAAGAA
GAGGGTATCTAGGCGTGGACGTGGCCGCTCTCGTTTTTCTAAGCGCCACCACACTGGGT
AACTGACTACAGTTGAGAAGGGGTGGACCGTAAACCACTGGCTGGCTGACTACAGCAAC
TGACTTTCCCGAGGCCACGGCGAGTAGCATCGAGGGTACAGGAAAGCTGGGACCATTGT
GTGACCAACTAATTTGGTTGCACTAGGGGAACCAATGGGGTGGTTTTTTTGTTGTGTGAA
ATACGGGGTCACCTCCCCCCACATACCTCTAAGGGCTTTTGAGCCTAGCGTTGGGCTACG
TTCTCGCATAAGGTCGGCTATACGACGTTTGTAGGGGGTAGTGCCAAACAACCCCTGAG
GTGACAGGTTCTGGTGGTGTTTAGTGAGCAGACATACAATAGACAGTGACAACATGGCT
TCAAGCCTAAAACAGGGAGTATCTCCCAAACTAAGGGATGTCATTCTTGTATCCAAAGA
CATTTTTTTAAAGATCCTTACCAACCAACTCGTGCAGTGTCATTGAATTGATGGTTCATG
TCTACGTTGTCGAAAGAGGCGTAGAATGACGGGTGAACCCATCGGACCTGCATGAACAT
GGAGAAGCCACCATGTTCATGATCGGGTGTGGCAAGAAGCCTTAATCCCTGGTACCCCA
ACCGAGCCAGGCTCGGCGGATCTGGGATTTTTGCTTTTCTCGACATGGCCTATCTAGCCA
CACCTGTGCTAGGAGTCGGTTCTCGCATTACCGCCTTAGATCGTACTATTGATGCTATTA
CGTTGAAACCTCGAATCGACCTCCAAGATGTGTATACAATTGATCCCACACTGACACTG
CGTCAGATAGACGTTTGCAATTCAGGGCTTGATCTACACTGGATTGCCATTCTCTCAAAG

TATTATGCAGGACGGCGTGCGCGTTCCATGTAAACCGGTCTCGACTATACGCCCGTTTTC
GGATC

1 to 31: recommended flanking region

32 to 115: spacer sequence (random, G:C = 0.5)

116 to 327: target sequence CAstV

328 to 332: spacer between target sequences (5 Ts)

333 to 515: target sequence ANV

516 to 520: spacer between target sequences (5 Ts)

521 to 771: target sequence IBV

772 to 776: spacer between target sequences (5 Ts)

777 to 973: target sequence AvRVA

974 to 978: spacer between target sequences (5 Ts)

979 to 1136: target sequence ARV

1137 to 1221: spacer sequence (random, G:C = 0.5)

1222-1250: recommended flanking sequence

1.2 Supplementary Figures

	UDLA 550	UDLA 551	UDLA 552	UDLA 583	UDLA 584	UDLA 585	UDLA 620	UDLA 621	MZ3673...	GU5854...	JF309115...	MT7897...	MT7897...	MW4012...	MW4012...	JQ94607...
UDLA 550		98.89%	98.34%	98.61%	98.61%	98.61%	98.34%	98.89%	91.21%	93.91%	91.97%	90.38%	91.21%	84.27%	92.74%	86.98%
UDLA 551	98.89%		98.89%	98.61%	99.17%	99.17%	98.89%	99.45%	91.48%	93.91%	91.97%	90.11%	90.93%	84.27%	93.15%	86.98%
UDLA 552	98.34%	98.89%		97.51%	99.17%	99.17%	99.45%	99.45%	90.38%	92.80%	90.86%	89.56%	89.84%	84.68%	91.94%	86.98%
UDLA 583	98.61%	98.61%	97.51%		97.78%	97.78%	97.51%	98.06%	91.48%	94.18%	92.24%	90.66%	90.93%	83.87%	93.15%	86.70%
UDLA 584	98.61%	99.17%	99.17%	97.78%		100%	99.72%	99.72%	90.66%	93.07%	91.14%	89.84%	90.66%	85.08%	92.34%	87.26%
UDLA 585	98.61%	99.17%	99.17%	97.78%	100%		99.72%	99.72%	90.66%	93.07%	91.14%	89.84%	90.66%	85.08%	92.34%	87.26%
UDLA 620	98.34%	98.89%	99.45%	97.51%	99.72%	99.72%		99.45%	90.38%	92.80%	90.86%	89.56%	90.38%	85.48%	91.94%	86.98%
UDLA 621	98.89%	99.45%	99.45%	98.06%	99.72%	99.72%	99.45%		90.93%	93.35%	91.41%	90.11%	90.38%	84.68%	92.74%	87.53%
MZ367372.1 BELGIUM	91.21%	91.48%	90.38%	91.48%	90.66%	90.66%	90.38%	90.93%		92.31%	90.66%	93.68%	93.96%	83.06%	95.16%	84.34%
GU585496.1 BRAZIL	93.91%	93.91%	92.80%	94.18%	93.07%	93.07%	92.80%	93.35%	92.31%		98.06%	92.86%	93.41%	83.47%	93.95%	86.70%
JF309115.1 BRAZIL	91.97%	91.97%	90.86%	92.24%	91.14%	91.14%	90.86%	91.41%	90.66%	98.06%		90.93%	91.48%	81.45%	91.94%	85.04%
MT789787.1 CANADA	90.38%	90.11%	89.56%	90.66%	89.84%	89.84%	89.56%	90.11%	93.68%	92.86%	90.93%		92.86%	83.06%	95.97%	84.34%
MT789786.1 CANADA	91.21%	90.93%	89.84%	90.93%	90.66%	90.66%	90.38%	90.38%	93.96%	93.41%	91.48%	92.86%		81.85%	93.55%	83.52%
MW401208.1 CHINA	84.27%	84.27%	84.68%	83.87%	85.08%	85.08%	85.48%	84.68%	83.06%	83.47%	81.45%	83.06%	81.85%		84.27%	88.71%
MW401211.1 CHINA	92.74%	93.15%	91.94%	93.15%	92.34%	92.34%	91.94%	92.74%	95.16%	93.95%	91.94%	95.97%	93.55%	84.27%		82.66%
JQ946070.1 CROATIA	86.98%	86.98%	86.98%	86.70%	87.26%	87.26%	86.98%	87.53%	84.34%	86.70%	85.04%	84.34%	83.52%	88.71%	82.66%	
JX083353.1 CROATIA	87.81%	87.81%	87.81%	87.53%	88.09%	88.09%	87.81%	88.37%	84.34%	86.70%	85.04%	83.52%	84.34%	89.11%	82.26%	96.95%
KC633178.1 INDIA	93.96%	92.86%	92.31%	93.41%	92.58%	92.58%	92.31%	92.86%	92.31%	93.96%	92.03%	94.51%	93.13%	83.06%	92.74%	85.44%
KU711026.1 INDIA	81.98%	82.69%	82.69%	82.33%	82.33%	82.33%	82.69%	82.69%	82.69%	83.04%	81.98%	82.33%	83.39%	87.50%	82.66%	86.93%
MN871904.1 IRAN	86.54%	85.99%	85.99%	86.26%	86.26%	86.26%	86.54%	85.99%	85.44%	85.71%	84.34%	83.79%	85.99%	85.08%	81.85%	84.62%
MN871905.1 IRAN	86.54%	85.99%	85.99%	86.26%	86.26%	86.26%	86.54%	85.99%	85.44%	85.71%	84.34%	83.79%	85.99%	85.08%	81.85%	84.62%
ON093075.1 IRAQ	82.25%	82.25%	81.97%	82.54%	81.41%	81.41%	81.69%	81.69%	82.12%	82.25%	80.56%	79.89%	81.84%	81.05%	79.03%	80.56%
JN048398.1 ITALY	85.67%	86.23%	85.67%	85.40%	85.95%	85.95%	86.23%	85.67%	85.16%	85.67%	84.02%	84.62%	85.71%	85.89%	85.08%	87.88%
MT491731.1 MALAYSIA	90.36%	90.08%	89.53%	90.08%	89.81%	89.81%	89.53%	90.08%	96.43%	90.91%	89.26%	93.41%	92.31%	81.85%	92.34%	83.47%
MT491732.1 MALAYSIA	90.11%	89.84%	89.29%	89.84%	89.56%	89.56%	89.29%	89.84%	96.15%	90.66%	89.01%	93.68%	92.58%	82.26%	92.74%	83.24%
MW684817.1 NETHERL...	92.03%	92.31%	91.21%	92.31%	91.48%	91.48%	91.21%	91.76%	97.80%	92.58%	90.93%	93.41%	93.68%	83.06%	94.35%	84.62%
MW684831.1 NETHERL...	91.46%	91.74%	90.63%	91.74%	90.91%	90.91%	90.63%	91.18%	98.90%	92.56%	90.91%	93.68%	93.41%	82.66%	94.76%	84.57%
KX779116.1 POLAND	82.83%	82.55%	82.27%	82.83%	82.55%	82.55%	82.27%	82.55%	85.44%	82.27%	80.61%	83.79%	82.14%	71.37%	81.05%	76.45%
KX779117.1 POLAND	79.78%	79.78%	79.50%	79.50%	79.78%	79.78%	79.50%	79.78%	78.02%	78.39%	76.45%	77.75%	78.57%	79.84%	72.58%	83.10%
OQ685946.1 TANZANIA	91.48%	91.76%	90.66%	91.76%	90.93%	90.93%	90.66%	91.21%	93.68%	90.93%	89.01%	92.03%	91.48%	83.06%	92.34%	83.52%
DQ324846.1 UNITED S...	85.44%	85.44%	85.44%	84.89%	86.26%	86.26%	85.99%	85.99%	84.07%	84.89%	82.97%	84.34%	84.62%	90.73%	82.66%	89.01%
JF414802.1 UNITED ST...	92.31%	92.58%	91.48%	92.86%	91.76%	91.76%	91.48%	92.03%	96.70%	93.68%	91.76%	96.15%	94.51%	83.47%	98.39%	84.07%
NC 024452.1 ChPV	51.37%	51.65%	51.10%	51.65%	51.37%	51.37%	51.37%	51.37%	52.75%	52.20%	51.92%	54.12%	53.30%	52.02%	53.63%	50.27%

	JX083353...	KC63317...	KU71102...	MN8719...	MN8719...	ON0930...	JN04839...	MT4917...	MT4917...	MW6848...	MW6848...	KX77911...	KX77911...	OQ6859...	DQ3248...	JF414802...	NC 0244...
UDLA 550	87.81%	93.96%	81.98%	86.54%	86.54%	82.25%	85.67%	90.36%	90.11%	92.03%	91.46%	82.83%	79.78%	91.48%	85.44%	92.31%	51.37%
UDLA 551	87.81%	92.86%	82.69%	85.99%	85.99%	82.25%	86.23%	90.08%	89.84%	92.31%	91.74%	82.55%	79.78%	91.76%	85.44%	92.58%	51.65%
UDLA 552	87.81%	92.31%	82.69%	85.99%	85.99%	81.97%	85.67%	89.53%	89.29%	91.21%	90.63%	82.27%	79.50%	90.66%	85.44%	91.48%	51.10%
UDLA 583	87.53%	93.41%	82.33%	86.26%	86.26%	82.54%	85.40%	90.08%	89.84%	92.31%	91.74%	82.83%	79.50%	91.76%	84.89%	92.86%	51.65%
UDLA 584	88.09%	92.58%	82.33%	86.26%	86.26%	81.41%	85.95%	89.81%	89.56%	91.48%	90.91%	82.55%	79.78%	90.93%	86.26%	91.76%	51.37%
UDLA 585	88.09%	92.58%	82.33%	86.26%	86.26%	81.41%	85.95%	89.81%	89.56%	91.48%	90.91%	82.55%	79.78%	90.93%	86.26%	91.76%	51.37%
UDLA 620	87.81%	92.31%	82.69%	86.54%	86.54%	81.69%	86.23%	89.53%	89.29%	91.21%	90.63%	82.27%	79.50%	90.66%	85.99%	91.48%	51.37%
UDLA 621	88.37%	92.86%	82.69%	85.99%	85.99%	81.69%	85.67%	90.08%	89.84%	91.76%	91.18%	82.55%	79.78%	91.21%	85.99%	92.03%	51.37%
MZ367372.1 BELGIUM	84.34%	92.31%	82.69%	85.44%	85.44%	82.12%	85.16%	96.43%	96.15%	97.80%	98.90%	85.44%	78.02%	93.68%	84.07%	96.70%	52.75%
GU585496.1 BRAZIL	86.70%	93.96%	83.04%	85.71%	85.71%	82.25%	85.67%	90.91%	90.66%	92.58%	92.56%	82.27%	78.39%	90.93%	84.89%	93.68%	52.20%
JF309115.1 BRAZIL	85.04%	92.03%	81.98%	84.34%	84.34%	80.56%	84.02%	89.26%	89.01%	90.93%	90.91%	80.61%	76.45%	89.01%	82.97%	91.76%	51.92%
MT789787.1 CANADA	83.52%	94.51%	82.33%	83.79%	83.79%	79.89%	84.62%	93.41%	93.68%	93.41%	93.68%	83.79%	77.75%	92.03%	84.34%	96.15%	54.12%
MT789786.1 CANADA	84.34%	93.13%	83.39%	85.99%	85.99%	81.84%	85.71%	92.31%	92.58%	93.68%	93.41%	82.14%	78.57%	91.48%	84.62%	94.51%	53.30%
MW401208.1 CHINA	89.11%	83.06%	87.50%	85.08%	85.08%	81.05%	85.89%	81.85%	82.26%	83.06%	82.66%	71.37%	79.84%	83.06%	90.73%	83.47%	52.02%
MW401211.1 CHINA	82.26%	92.74%	82.66%	81.85%	81.85%	79.03%	85.08%	92.34%	92.74%	94.35%	94.76%	81.05%	72.58%	92.34%	82.66%	98.39%	53.63%
JQ946070.1 CROATIA	96.95%	85.44%	86.93%	84.62%	84.62%	80.56%	87.88%	83.47%	83.24%	84.62%	84.57%	76.45%	83.10%	83.52%	89.01%	84.07%	50.27%
JX083353.1 CROATIA		85.44%	86.93%	85.16%	85.16%	81.13%	87.05%	83.47%	83.24%	84.62%	84.57%	77.01%	85.04%	84.07%	88.74%	84.07%	50.55%
KC633178.1 INDIA	85.44%		83.75%	85.99%	85.99%	81.01%	85.99%	91.76%	92.03%	92.86%	92.86%	83.24%	78.30%	92.03%	84.62%	94.23%	52.20%
KU711026.1 INDIA	86.93%	83.75%		84.45%	84.45%	79.86%	86.57%	80.92%	80.92%	81.98%	82.33%	71.02%	77.74%	81.63%	86.22%	82.33%	50.53%
MN871904.1 IRAN	85.16%	85.99%	84.45%		100%	86.59%	86.26%	85.99%	86.26%	85.16%	85.44%	75.55%	76.92%	83.52%	85.99%	84.34%	51.37%
MN871905.1 IRAN	85.16%	85.99%	84.45%	100%		86.59%	86.26%	85.99%	86.26%	85.16%	85.44%	75.55%	76.92%	83.52%	85.99%	84.34%	51.37%
ON093075.1 IRAQ	81.13%	81.01%	79.86%	86.59%	86.59%		82.35%	81.51%	81.28%	82.40%	82.07%	82.69%	84.48%	79.61%	80.45%	82.68%	47.77%
JN048398.1 ITALY	87.05%	85.99%	86.57%	86.26%	86.26%	82.35%		84.85%	84.07%	84.89%	84.85%	77.41%	78.24%	83.79%	85.44%	86.26%	52.47%
MT491731.1 MALAYSIA	83.47%	91.76%	80.92%	85.99%	85.99%	81.51%	84.85%		98.90%	96.15%	96.42%	85.40%	76.86%	93.41%	84.34%	94.78%	51.92%
MT491732.1 MALAYSIA	83.24%	92.03%	80.92%	86.26%	86.26%	81.28%	84.07%	98.90%		96.15%	96.70%	85.16%	76.65%	93.13%	84.07%	94.51%	52.47%
MW684817.1 NETHERL...	84.62%	92.86%	81.98%	85.16%	85.16%	82.40%	84.89%	96.15%	96.15%		97.53%	85.71%	78.30%	93.96%	84.62%	96.43%	53.02%
MW684831.1 NETHERL...	84.57%	92.86%	82.33%	85.44%	85.44%	82.07%	84.85%	96.42%	96.70%	97.53%		85.67%	78.24%	93.13%	84.07%	96.15%	52.75%
KX779116.1 POLAND	77.01%	83.24%	71.02%	75.55%	75.55%	82.69%	77.41%	85.40%	85.16%	85.71%	85.67%		84.85%	83.79%	74.45%	85.99%	48.08%
KX779117.1 POLAND	85.04%	78.30%	77.74%	76.92%	76.92%	84.48%	78.24%	76.86%	76.65%	78.30%	78.24%	84.85%		78.85%	81.59%	79.12%	46.43%
OQ685946.1 TANZANIA	84.07%	92.03%	81.63%	83.52%	83.52%	79.61%	83.79%	93.41%	93.13%	93.96%	93.13%	83.79%	78.85%		83.79%	94.23%	52.47%
DQ324846.1 UNITED S...	88.74%	84.62%	86.22%	85.99%	85.99%	80.45%	85.44%	84.34%	84.07%	84.62%	84.07%	74.45%	81.59%	83.79%		84.07%	49.45%
JF414802.1 UNITED ST...	84.07%	94.23%	82.33%	84.34%	84.34%	82.68%	86.26%	94.78%	94.51%	96.43%	96.15%	85.99%	79.12%	94.23%	84.07%		53.30%
NC 024452.1 ChPV	50.55%	52.20%	50.53%	51.37%	51.37%	47.77%	52.47%	51.92%	52.47%	53.02%	52.75%	48.08%	46.43%	52.47%	49.45%	53.30%	

Supplementary Figure 1. Comparison of the nucleotide identities of the sequences of Ecuadorian samples of ANV with other sequences of this virus.

	UDLA 652	UDLA 554	UDLA 557	UDLA 560	UDLA 563	UDLA 564	UDLA 561	UDLA 562	HM0178...	HM0178...	MF50988...	MF68340...	MZ1505...
UDLA 652		99.58%	97.46%	99.58%	97.46%	98.31%	99.37%	99.58%	92.24%	93.07%	89.01%	94.65%	94.29%
UDLA 554	99.58%		97.89%	100%	97.89%	98.73%	99.79%	100%	92.24%	93.07%	88.58%	94.65%	94.29%
UDLA 557	97.46%	97.89%		97.89%	96.19%	97.04%	97.67%	97.89%	92.24%	93.63%	88.37%	93.54%	93.45%
UDLA 560	99.58%	100%	97.89%		97.89%	98.73%	99.79%	100%	92.24%	93.07%	88.58%	94.65%	94.29%
UDLA 563	97.46%	97.89%	96.19%	97.89%		99.15%	97.67%	97.89%	91.69%	93.07%	87.74%	92.87%	93.23%
UDLA 564	98.31%	98.73%	97.04%	98.73%	99.15%		98.52%	98.73%	91.97%	92.80%	88.58%	93.54%	93.87%
UDLA 561	99.37%	99.79%	97.67%	99.79%	97.67%	98.52%		99.79%	92.24%	93.07%	88.37%	94.65%	94.08%
UDLA 562	99.58%	100%	97.89%	100%	97.89%	98.73%	99.79%		92.24%	93.07%	88.58%	94.65%	94.29%
HM017852.1 AUSTRALIA	92.24%	92.24%	92.24%	92.24%	91.69%	91.97%	92.24%	92.24%		96.12%	89.47%	90.30%	91.69%
HM017853.1 AUSTRALIA	93.07%	93.07%	93.63%	93.07%	93.07%	92.80%	93.07%	93.07%	96.12%		88.37%	90.58%	91.97%
MF509886.1 BRAZIL	89.01%	88.58%	88.37%	88.58%	87.74%	88.58%	88.37%	88.58%	89.47%	88.37%		87.31%	87.32%
MF683401.1 BRAZIL	94.65%	94.65%	93.54%	94.65%	92.87%	93.54%	94.65%	94.65%	90.30%	90.58%	87.31%		90.87%
MZ150559.1 CHINA	94.29%	94.29%	93.45%	94.29%	93.23%	93.87%	94.08%	94.29%	91.69%	91.97%	87.32%	90.87%	
MW522993.1 CHINA	91.88%	91.67%	91.24%	91.67%	90.17%	90.81%	91.45%	91.67%	89.47%	88.64%	86.11%	88.86%	89.10%
JN048379.1 ITALY	91.75%	92.18%	91.54%	92.18%	91.12%	91.75%	91.97%	92.18%	91.14%	91.41%	87.32%	89.31%	92.18%
JN048380.1 ITALY	91.75%	92.18%	91.54%	92.18%	91.12%	91.75%	91.97%	92.18%	91.14%	91.41%	87.32%	89.31%	92.18%
OP967012.1 INDIA	91.54%	91.79%	92.05%	91.79%	91.28%	91.54%	91.79%	91.79%	89.09%	89.39%	85.13%	89.45%	90.77%
OP967004.1 INDIA	93.85%	94.10%	93.59%	94.10%	92.56%	92.82%	94.10%	94.10%	90.61%	90.91%	85.64%	91.29%	92.56%
MT380201.1 ISRAEL	96.02%	96.02%	95.41%	96.02%	94.19%	94.19%	96.02%	96.02%	90.85%	91.18%	88.69%	94.50%	95.41%
NC_003790.1 JAPAN	88.79%	88.37%	88.16%	88.37%	86.68%	87.53%	88.16%	88.37%	89.75%	88.09%	94.71%	87.75%	87.95%
KC593398.1 SOUTH KO...	94.19%	94.19%	93.58%	94.19%	93.27%	93.58%	94.19%	94.19%	91.69%	91.36%	86.24%	93.88%	92.35%
KC593401.1 SOUTH KO...	93.58%	93.58%	93.58%	93.58%	92.66%	92.97%	93.58%	93.58%	90.37%	90.70%	85.32%	91.74%	92.05%
OQ685945.1 TANZANIA	91.33%	91.33%	90.49%	91.33%	89.85%	90.49%	91.12%	91.33%	91.14%	91.41%	87.10%	90.20%	90.49%
HQ188698.1 UNITED S...	95.35%	95.60%	96.09%	95.60%	94.62%	94.87%	95.60%	95.60%	91.98%	92.84%	88.75%	92.71%	92.91%
DQ324827.1 UNITED S...	93.23%	92.81%	92.81%	92.81%	91.33%	91.97%	92.60%	92.81%	91.14%	91.97%	87.10%	91.98%	91.97%
MT585643.1 UNITED KI...	93.23%	93.23%	93.23%	93.23%	91.33%	91.97%	93.02%	93.23%	92.24%	92.52%	88.16%	92.65%	93.87%
NC_024452.1 ChPV	53.28%	52.85%	52.22%	52.85%	53.28%	53.49%	52.64%	52.85%	51.52%	52.08%	51.80%	52.34%	51.37%

	MW5229...	JN04837...	JN04838...	OP96701...	OP96700...	MT3802...	NC_0037...	KC59339...	KC59340...	OQ6859...	HQ1886...	DQ3248...	MT5856...	NC_0244...
UDLA 652	91.88%	91.75%	91.75%	91.54%	93.85%	96.02%	88.79%	94.19%	93.58%	91.33%	95.35%	93.23%	93.23%	53.28%
UDLA 554	91.67%	92.18%	92.18%	91.79%	94.10%	96.02%	88.37%	94.19%	93.58%	91.33%	95.60%	92.81%	93.23%	52.85%
UDLA 557	91.24%	91.54%	91.54%	92.05%	93.59%	95.41%	88.16%	93.58%	93.58%	90.49%	96.09%	92.81%	93.23%	52.22%
UDLA 560	91.67%	92.18%	92.18%	91.79%	94.10%	96.02%	88.37%	94.19%	93.58%	91.33%	95.60%	92.81%	93.23%	52.85%
UDLA 563	90.17%	91.12%	91.12%	91.28%	92.56%	94.19%	86.68%	93.27%	92.66%	89.85%	94.62%	91.33%	91.33%	53.28%
UDLA 564	90.81%	91.75%	91.75%	91.54%	92.82%	94.19%	87.53%	93.58%	92.97%	90.49%	94.87%	91.97%	91.97%	53.49%
UDLA 561	91.45%	91.97%	91.97%	91.79%	94.10%	96.02%	88.16%	94.19%	93.58%	91.12%	95.60%	92.60%	93.02%	52.64%
UDLA 562	91.67%	92.18%	92.18%	91.79%	94.10%	96.02%	88.37%	94.19%	93.58%	91.33%	95.60%	92.81%	93.23%	52.85%
HM017852.1 AUSTRALIA	89.47%	91.14%	91.14%	89.09%	90.61%	90.85%	89.75%	91.69%	90.37%	91.14%	91.98%	91.14%	92.24%	51.52%
HM017853.1 AUSTRALIA	88.64%	91.41%	91.41%	89.39%	90.91%	91.18%	88.09%	91.36%	90.70%	91.41%	92.84%	91.97%	92.52%	52.08%
MF509886.1 BRAZIL	86.11%	87.32%	87.32%	85.13%	85.64%	88.69%	94.71%	86.24%	85.32%	87.10%	88.75%	87.10%	88.16%	51.80%
MF683401.1 BRAZIL	88.86%	89.31%	89.31%	89.45%	91.29%	94.50%	87.75%	93.88%	91.74%	90.20%	92.71%	91.98%	92.65%	52.34%
MZ150559.1 CHINA	89.10%	92.18%	92.18%	90.77%	92.56%	95.41%	87.95%	92.35%	92.05%	90.49%	92.91%	91.97%	93.87%	51.37%
MW522993.1 CHINA		89.10%	89.10%	88.08%	89.38%	89.60%	87.61%	88.07%	89.91%	88.25%	92.35%	89.96%	89.96%	50.85%
JN048379.1 ITALY	89.10%		100%	90.00%	91.79%	90.83%	86.26%	89.60%	92.66%	90.27%	91.93%	91.33%	92.39%	50.32%
JN048380.1 ITALY	89.10%	100%		90.00%	91.79%	90.83%	86.26%	89.60%	92.66%	90.27%	91.93%	91.33%	92.39%	50.32%
OP967012.1 INDIA	88.08%	90.00%	90.00%		96.15%	89.45%	85.38%	91.44%	91.74%	91.54%	92.56%	90.26%	91.79%	51.79%
OP967004.1 INDIA	89.38%	91.79%	91.79%	96.15%		93.09%	86.41%	91.44%	92.35%	92.31%	94.36%	92.05%	94.10%	52.31%
MT380201.1 ISRAEL	89.60%	90.83%	90.83%	89.45%	93.09%		89.91%	92.68%	92.28%	90.52%	93.20%	93.88%	95.11%	49.54%
NC_003790.1 JAPAN	87.61%	86.26%	86.26%	85.38%	86.41%	89.91%		86.24%	86.85%	86.47%	88.75%	87.53%	88.79%	51.37%
KC593398.1 SOUTH KO...	88.07%	89.60%	89.60%	91.44%	91.44%	92.68%	86.24%		92.05%	90.21%	93.58%	93.88%	93.27%	52.29%
KC593401.1 SOUTH KO...	89.91%	92.66%	92.66%	91.74%	92.35%	92.28%	86.85%	92.05%		89.91%	93.27%	92.97%	92.35%	52.29%
OQ685945.1 TANZANIA	88.25%	90.27%	90.27%	91.54%	92.31%	90.52%	86.47%	90.21%	89.91%		91.69%	89.64%	90.91%	52.01%
HQ188698.1 UNITED S...	92.35%	91.93%	91.93%	92.56%	94.36%	93.20%	88.75%	93.58%	93.27%	91.69%		95.35%	95.60%	52.57%
DQ324827.1 UNITED S...	89.96%	91.33%	91.33%	90.26%	92.05%	93.88%	87.53%	93.88%	92.97%	89.64%	95.35%		94.50%	52.01%
MT585643.1 UNITED KI...	89.96%	92.39%	92.39%	91.79%	94.10%	95.11%	88.79%	93.27%	92.35%	90.91%	95.60%	94.50%		50.95%
NC_024452.1 ChPV	50.85%	50.32%	50.32%	51.79%	52.31%	49.54%	51.37%	52.29%	52.29%	52.01%	52.57%	52.01%	50.95%	

Supplementary Figure 2. Comparison of the nucleotide identities of the sequences of Ecuadorian samples of CAstV with other sequences of this virus.

	UDLA 578	UDLA 501	UDLA 505	UDLA 573	UDLA 566	UDLA 503	AF093795 ...	AJ618985 G...	JQ739375 ...	Z83975 GI-...	UDLA 525	UDLA 569	AY561711 ...	FJ888351 G...	KJ200289 G...	KF809793 ...
UDLA 578		99.78%	99.78%	99.78%	99.78%	99.78%	96.07%	97.38%	99.78%	99.78%	77.95%	77.95%	79.04%	77.73%	77.95%	77.95%
UDLA 501	99.78%		100%	100%	100%	100%	96.29%	97.60%	100%	100%	77.73%	77.73%	78.82%	77.51%	77.73%	77.73%
UDLA 505	99.78%	100%		100%	100%	100%	96.29%	97.60%	100%	100%	77.73%	77.73%	78.82%	77.51%	77.73%	77.73%
UDLA 573	99.78%	100%	100%		100%	100%	96.29%	97.60%	100%	100%	77.73%	77.73%	78.82%	77.51%	77.73%	77.73%
UDLA 566	99.78%	100%	100%	100%		100%	96.29%	97.60%	100%	100%	77.73%	77.73%	78.82%	77.51%	77.73%	77.73%
UDLA 503	99.78%	100%	100%	100%	100%		96.29%	97.60%	100%	100%	77.73%	77.73%	78.82%	77.51%	77.73%	77.73%
AF093795 GI-13	96.07%	96.29%	96.29%	96.29%	96.29%	96.29%		95.67%	95.49%	95.36%	77.73%	77.73%	77.54%	77.19%	77.05%	74.39%
AJ618985 GI-13	97.38%	97.60%	97.60%	97.60%	97.60%	97.60%	95.67%		96.35%	96.54%	77.73%	77.73%	77.61%	77.25%	77.11%	74.39%
JQ739375 GI-13	99.78%	100%	100%	100%	100%	100%	95.49%	96.35%		99.51%	77.73%	77.73%	78.29%	77.87%	77.73%	74.91%
Z83975 GI-13	99.78%	100%	100%	100%	100%	100%	95.36%	96.54%	99.51%		77.73%	77.73%	78.22%	77.68%	77.54%	74.94%
UDLA 525	77.95%	77.73%	77.73%	77.73%	77.73%	77.73%	77.73%	77.73%	77.73%	77.73%		100%	98.47%	99.78%	100%	100%
UDLA 569	77.95%	77.73%	77.73%	77.73%	77.73%	77.73%	77.73%	77.73%	77.73%	77.73%	100%		98.47%	99.78%	100%	100%
AY561711 GI-1	79.04%	78.82%	78.82%	78.82%	78.82%	78.82%	77.54%	77.61%	78.29%	78.22%	98.47%	98.47%		97.58%	97.44%	94.55%
FJ888351 GI-1	77.73%	77.51%	77.51%	77.51%	77.51%	77.51%	77.19%	77.25%	77.87%	77.68%	99.78%	99.78%	97.58%		99.69%	96.41%
KJ200289 GI-1	77.95%	77.73%	77.73%	77.73%	77.73%	77.73%	77.05%	77.11%	77.73%	77.54%	100%	100%	97.44%	99.69%		96.70%
KF809793 GI-1	77.95%	77.73%	77.73%	77.73%	77.73%	77.73%	74.39%	74.39%	74.91%	74.94%	100%	100%	94.55%	96.41%	96.70%	

Supplementary Figure 3. Comparison of the nucleotide identities of the sequences of Ecuadorian samples of IBV with other sequences of this virus.

	MK246989 ...	AF354224 ...	L39002 CA...	AF204950 ...	UDLA 511	UDLA 570	AF004857 ...	UDLA 502	UDLA 549	UDLA 551	UDLA 567	OR815314 ...	UDLA 572	KJ879625 U...	KX855920 ...
MK246989 UNITED STA...		74.292%	76.331%	77.123%	75.232%	75.095%	77.237%	75.232%	75.397%	75.550%	75.448%	59.230%	56.026%	59.003%	58.522%
AF354224 NETHERLAN...	74.292%		76.656%	77.166%	74.776%	75.000%	77.574%	75.096%	74.669%	74.903%	75.062%	59.934%	56.958%	61.277%	59.417%
L39002 CANADA	76.331%	76.656%		97.554%	97.695%	98.153%	98.434%	98.203%	98.214%	98.318%	98.379%	58.946%	54.591%	58.617%	57.511%
AF204950 CHINA	77.123%	77.166%	97.554%		98.464%	99.015%	98.981%	99.230%	99.008%	99.094%	99.002%	59.056%	55.093%	58.617%	57.623%
UDLA 511	75.232%	74.776%	97.695%	98.464%		99.098%	99.232%	99.230%	99.932%	99.866%	99.355%	56.978%	55.380%	56.210%	56.082%
UDLA 570	75.095%	75.000%	98.153%	99.015%	99.098%		99.877%	99.871%	99.669%	99.741%	99.751%	57.143%	55.380%	56.773%	56.773%
AF004857 UNITED STA...	77.237%	77.574%	98.434%	98.981%	99.232%	99.877%		100%	99.802%	99.871%	99.875%	59.385%	55.237%	58.830%	57.848%
UDLA 502	75.232%	75.096%	98.203%	99.230%	99.230%	99.871%	100%		99.795%	99.866%	99.871%	56.996%	55.237%	56.483%	56.483%
UDLA 549	75.397%	74.669%	98.214%	99.008%	99.932%	99.669%	99.802%	99.795%		99.934%	99.934%	56.151%	55.452%	56.283%	55.886%
UDLA 551	75.550%	74.903%	98.318%	99.094%	99.866%	99.741%	99.871%	99.866%	99.934%		100%	56.792%	55.380%	56.533%	56.404%
UDLA 567	75.448%	75.062%	98.379%	99.002%	99.355%	99.751%	99.875%	99.871%	99.934%	100%		56.983%	55.380%	56.733%	56.608%
OR815314 UNITED STA...	59.230%	59.934%	58.946%	59.056%	56.978%	57.143%	59.385%	56.996%	56.151%	56.792%	56.983%		87.374%	67.618%	65.629%
UDLA 572	56.026%	56.958%	54.591%	55.093%	55.380%	55.380%	55.237%	55.237%	55.452%	55.380%	55.380%	87.374%		66.643%	64.562%
KJ879625 UNITED STAT...	59.003%	61.277%	58.617%	58.617%	56.210%	56.773%	58.830%	56.483%	56.283%	56.533%	56.733%	67.618%	66.643%		80.493%
KX855920 CANADA	58.522%	59.417%	57.511%	57.623%	56.082%	56.773%	57.848%	56.483%	55.886%	56.404%	56.608%	65.629%	64.562%	80.493%	

Supplementary Figure 4. Comparison of the nucleotide identities of the sequences of Ecuadorian samples of ARV with other sequences of this virus.

	MF683405....	UDLA 551	MN365943....	OM469201....	MF683406....	KT347546....	UDLA 550	UDLA 599	UDLA 147	UDLA 515	UDLA 572	LK932180.1...	LK932186.1...	KU372509....	AB009627....
MF683405.1 BRAZIL		88.735%	89.723%	90.316%	38.342%	38.302%	38.123%	38.039%	37.992%	38.039%	37.965%	39.157%	36.293%	37.037%	34.276%
UDLA 551	88.735%		91.640%	90.454%	35.800%	36.977%	37.727%	37.094%	37.227%	37.209%	37.307%	39.068%	36.554%	35.101%	33.195%
MN365943.1 GERMANY	89.723%	91.640%		92.902%	35.600%	36.013%	36.970%	36.321%	36.449%	36.434%	36.378%	38.103%	35.509%	34.552%	32.775%
OM469201.1 SWITZERL...	90.316%	90.454%	92.902%		36.800%	36.495%	37.424%	36.476%	36.604%	36.589%	36.533%	38.264%	36.031%	36.015%	33.380%
MF683406.1 BRAZIL	38.342%	35.800%	35.600%	36.800%		88.454%	88.351%	88.454%	88.454%	88.454%	88.454%	89.485%	90.110%	76.190%	72.746%
KT347546.1 PERU	38.302%	36.977%	36.013%	36.495%	88.454%		94.802%	97.190%	97.176%	97.181%	97.181%	88.851%	90.909%	75.888%	74.754%
UDLA 550	38.123%	37.727%	36.970%	37.424%	88.351%	94.802%		97.857%	98.000%	97.850%	97.854%	89.868%	90.508%	75.794%	74.381%
UDLA 599	38.039%	37.094%	36.321%	36.476%	88.454%	97.190%	97.857%		100%	100%	100%	90.610%	91.711%	75.888%	76.303%
UDLA 147	37.992%	37.227%	36.449%	36.604%	88.454%	97.176%	98.000%	100%		100%	100%	90.610%	91.711%	75.888%	76.115%
UDLA 515	38.039%	37.209%	36.434%	36.589%	88.454%	97.181%	97.850%	100%	100%		100%	90.610%	91.711%	75.888%	76.228%
UDLA 572	37.965%	37.307%	36.378%	36.533%	88.454%	97.181%	97.854%	100%	100%	100%		90.610%	91.711%	75.888%	76.266%
LK932180.1 NIGERIA	39.157%	39.068%	38.103%	38.264%	89.485%	88.851%	89.868%	90.610%	90.610%	90.610%	90.610%		94.920%	76.262%	75.738%
LK932186.1 NIGERIA	36.293%	36.554%	35.509%	36.031%	90.110%	90.909%	90.508%	91.711%	91.711%	91.711%	91.711%	94.920%		75.740%	74.866%
KU372509.1 NIGERIA	37.037%	35.101%	34.552%	36.015%	76.190%	75.888%	75.794%	75.888%	75.888%	75.888%	75.888%	76.262%	75.740%		76.449%
AB009627.1 JAPON	34.276%	33.195%	32.775%	33.380%	72.746%	74.754%	74.381%	76.303%	76.115%	76.228%	76.266%	75.738%	74.866%	76.449%	

Supplementary Figure 5. Comparison of the nucleotide identities of the sequences of Ecuadorian samples of AvRVA with other sequences of this virus.

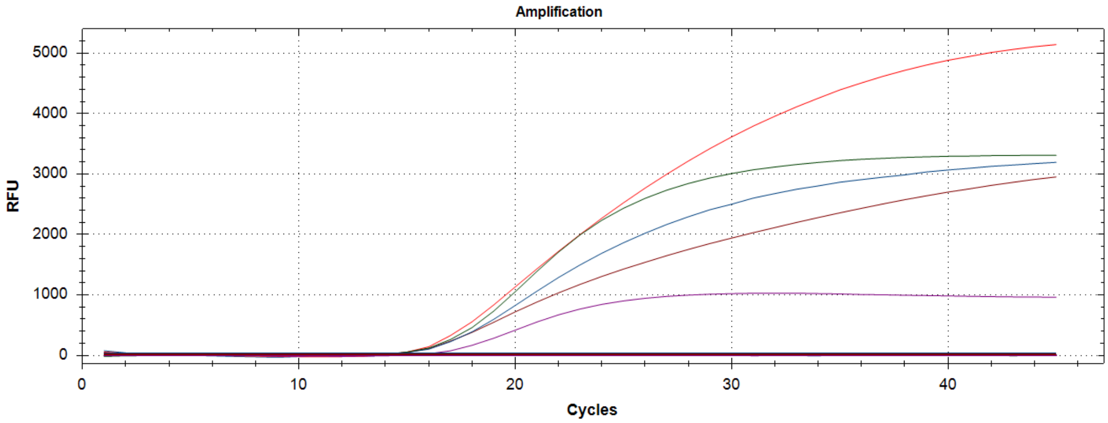

Supplementary Figure 6. Positive Control of the specificity assay. Red color for IBV amplification plot, green color for ANV amplification plot, blue color for CAstV amplification plot, brown color for ARV amplification plot and purple color for AvRVA amplification plot.

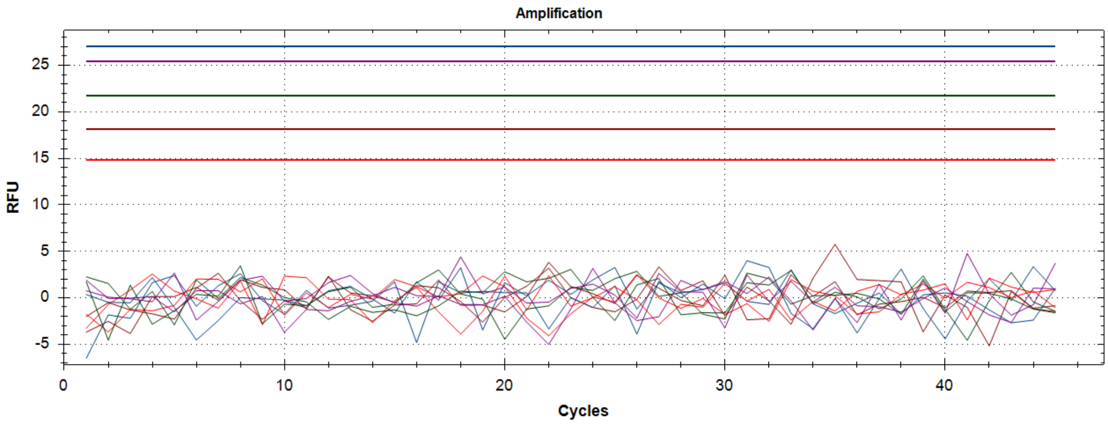

Supplementary Figure 7. ChPV Control of the specificity assay. Red color for IBV non-amplification plot, green color for ANV non-amplification plot, blue color for CAstV non-

amplification, brown color for ARV non-amplification plot and purple color for AvRVA non-amplification plot.

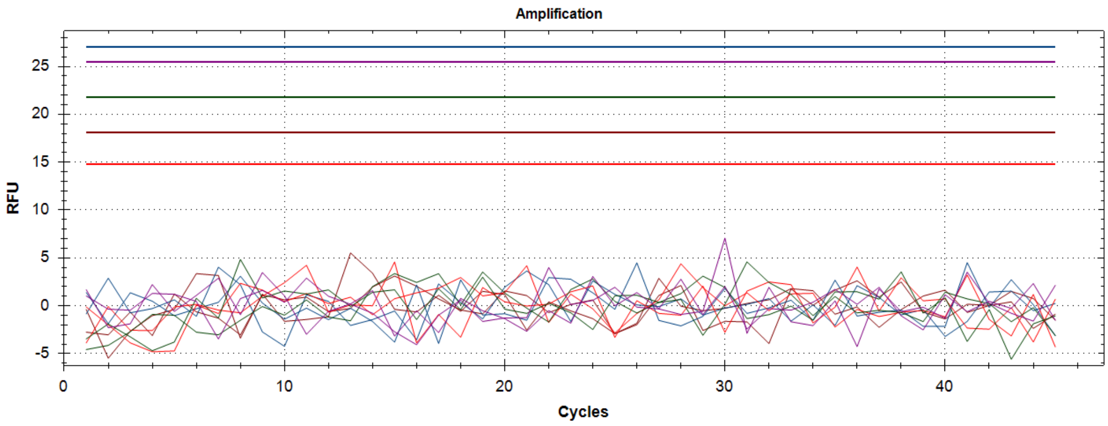

Supplementary Figure 8. aMPV-A Control of the specificity assay. Red color for IBV non-amplification plot, green color for ANV non-amplification plot, blue color for CAstV non-amplification, brown color for ARV non-amplification plot and purple color for AvRVA non-amplification plot.

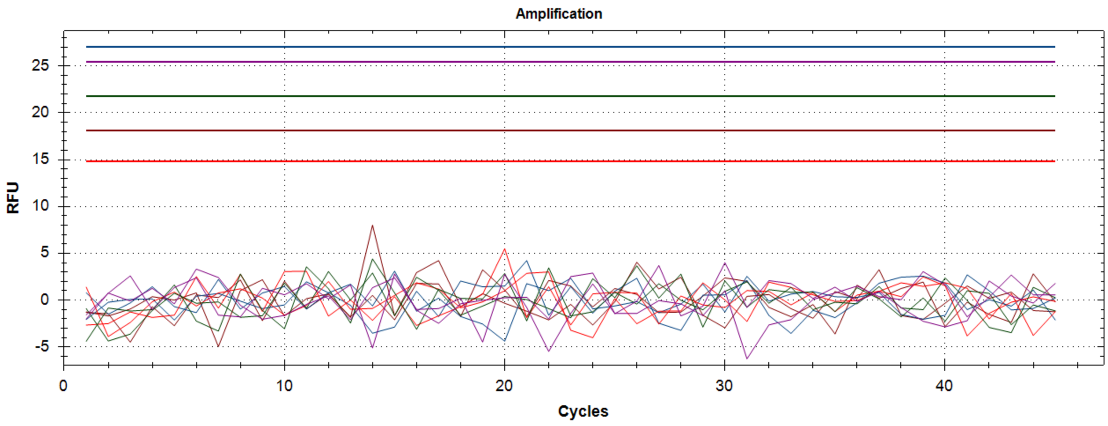

Supplementary Figure 9. aMPV-B Control of the specificity assay. Red color for IBV non-amplification plot, green color for ANV non-amplification plot, blue color for CAstV non-

amplification, brown color for ARV non-amplification plot and purple color for AvRVA non-amplification plot.

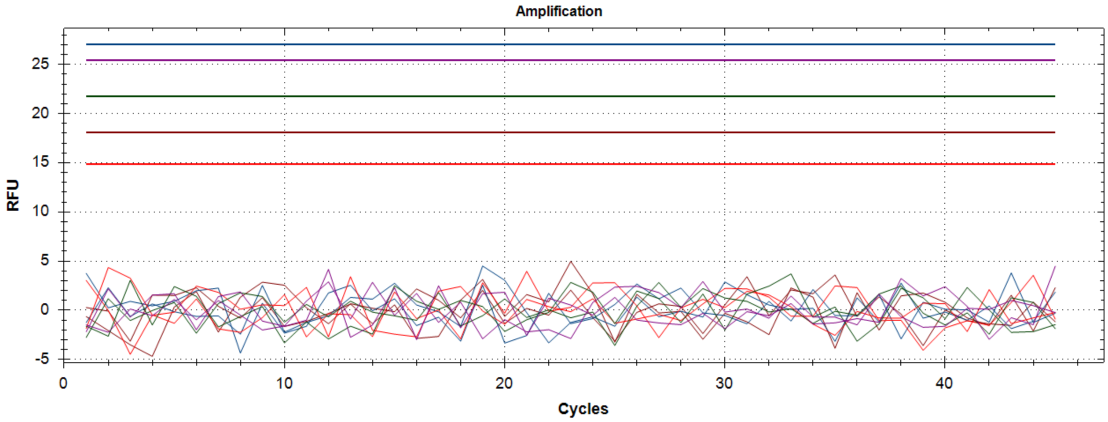

Supplementary Figure 10. FAdV-1 Control of the specificity assay. Red color for IBV non-amplification plot, green color for ANV non-amplification plot, blue color for CAstV non-amplification, brown color for ARV non-amplification plot and purple color for AvRVA non-amplification plot.
